# Do Larger Earned Income Tax Credit and Supplemental Nutrition Assistance Program Benefits Create Complementary Effects on Child Development?

**DOI:** 10.1007/s11113-025-09985-9

**Published:** 2026-01-22

**Authors:** Youngjin Stephanie Hong

**Affiliations:** Department of Population Health Sciences, University of Wisconsin-Madison, Madison, WI USA

**Keywords:** Early cognitive development, Poverty, EITC, SNAP, Safety net, Multiple program participation, Working poor

## Abstract

Poverty is particularly concerning during early childhood and the early school years, as it can negatively impact child development both in the short and long term. To alleviate economic hardship, the U.S. government provides a patchwork of income support policies. This paper examines two of the largest programs, the Supplemental Nutrition Assistance Program (SNAP) and the Earned Income Tax Credit (EITC), which low-income working families often receive simultaneously. This paper is the first to explore whether these benefits interact to influence children's early cognitive development in families receiving both programs. To address endogeneity of program benefits, I use a two-way (child and year) fixed effects model to leverage the variation in the maximum federal and state EITC benefits stemming from changes in the number of children and state EITC policies, as well as the variation in SNAP purchasing power driven by local food prices over time within each child, rather than actual benefit amounts. Using data from the Early Childhood Longitudinal Study-Birth Cohort (which tracks a nationally representative 2001 birth cohort through the kindergarten-entry period), I find new population-level evidence that EITC benefits are effective at improving early math and reading skills when coupled with greater SNAP purchasing power, and vice versa (sample size = 1300). Suggestive evidence is provided on the mechanisms underlying such complementary effects on early cognitive outcomes. The findings highlight the importance of enhancing the reach and generosity of both programs.

**Supplementary Information:**

The online version contains supplementary material available at 10.1007/s11113-025-09985-9.

## Introduction

The U.S. government provides multiple public programs to reduce poverty and economic hardships. Since the 1990s, in-kind food assistance and refundable tax credits have become particularly essential for low-income families – especially the working poor – as the U.S. social safety net has shifted away from cash-based assistance (Gilbert, 2009; Hardy et al., 2018). As of writing, the Supplemental Nutrition Assistance Program (SNAP), a monthly benefit that allows low-income populations to purchase food, is the largest means-tested income support (cash or near-cash) program for families with children (United States Department of Agriculture [USDA], 2021). By offsetting food costs, SNAP may increase a household’s consumption of other needs by freeing up economic resources that would otherwise be spent on food (if the household is inframarginal, i.e., spends more on food than the amount of SNAP benefits). Following SNAP, the Earned Income Tax Credit (EITC) is the second largest means-tested income support program for families with children (Internal Revenue Service [IRS], 2021). The EITC is a refundable tax credit that provides lumpsum cash benefits to low-to-moderate-income working populations after taxes are filed. By design, the EITC is less likely to serve the lowest-income families (e.g., in deep poverty) (Hoynes et al., 2018).

Given that the U.S. social safety net programs are multidimensional and overlapping in their eligibility criteria, there have been growing interests in potential complementary effects that multiple programs may have on child development (Jackson, & Finelli, 2023). In this paper, I attempt to provide a more comprehensive understanding of how the two largest U.S. income support programs influence child development in low-income families, by examining their interaction effects on early cognitive development in preschool to kindergarten-entry period. Specifically, I examine whether greater EITC benefits are more effective at improving early reading and early math skills when they are coupled with larger SNAP values, and vice versa, among families who likely receive both benefits. The populations who jointly receive them comprise low-income working families, who have consistently represented a significant proportion, over 70%, of the low-income population in the U.S. (Urban Institute, 2009; Roberts et al., 2013).

Joint participation in SNAP and EITC is quite a common phenomenon. Based on the 2008–2009 Survey of Income and Program Participation data, Moffitt (2020) shows that among the major tax and transfer programs, the most common receipt among SNAP participants is of EITC benefits, excluding Medicaid. Among non-elderly, non-disabled SNAP families, 53% of them – and 95% of those families with incomes between 50 and 100% of the poverty line – likely received EITC benefits.^1^ Similarly, in more recent 2019 data, EITC and SNAP represented one of the most common program bundles received by people who participate in multiple safety net programs (Macartney, & Ghertner, 2023). EITC and SNAP are also unique bundles, since participating in one program does not *directly* affect one’s eligibility and benefit amounts for the other program. That is because refundable tax credits are not generally countable income and resources considered in SNAP eligibility and benefit determination (Moffitt, 2020) and SNAP benefits are not earned income and therefore are not included in calculation of EITC eligibility and benefit amounts. However, it is still possible that EITC and SNAP affect one another *indirectly* through labor supply changes, which I discuss more in-depth in the discussion section.

While previous studies have examined how each program, SNAP and EITC, influences school-age children’s test scores and/or adult health and economic outcomes (e.g., Dahl et al., 2012; Gassman-Pines et al., 2018; Hoynes et al., 2016), there is limited evidence on their effects on early cognitive development. Understanding these dynamics is important because early childhood and kindergarten-entry are developmental stages especially sensitive to family income (National Academies of Sciences, Engineering, and Medicine [NASEM], 2019). Poverty in these periods can have lasting negative consequences for children’s development (NASEM, 2019; Duncan et al., 2010), creating large income-based gaps in math, literacy, and language even before they enter school (Waldfogel et al., 2011). Such school readiness skills can shape children’s future, as prior research has found early cognitive development is a particularly strong predictor of later academic achievement (Braak et al., 2022; Rabiner et al., 2016), educational attainment and income at age 30 (Feinstein, & Duckworth, 2006), and potentially health (La-Scherban et al., 2014), compared to other developmental domains. Understanding the effects of SNAP and EITC on early cognitive development would also advance our knowledge of how their medium- and long-term benefits are realized.

Furthermore, there is no research to date that examined the interaction effects of EITC and SNAP benefits on child development. As evidence suggests families may not treat those benefits as fungible (i.e., totally interchangeable) (e.g., Hastings et al., 2018; Sykes et al., 2015), they may affect different domains of child development and in turn produce complementary effects. For example, children who experience improvements in nutrition from SNAP benefits may derive greater returns from EITC investments in creating developmentally enriching home environments. Furthermore, when parents have greater SNAP benefits at hand, they may have greater economic foundation to spend a larger portion of their EITC benefits for children, compared to otherwise similar families with smaller SNAP benefits. As such, simply evaluating the EITC and SNAP benefits separately could give us an incomplete picture of their impacts on child development.

To reduce bias in estimating the EITC benefit level, I measure it as the maximum federal and state EITC benefits a child’s family could receive, given the year, state of residence, and number of children in the household. This approach allows me to leverage the EITC variation coming from changes in the number of children and from state EITC expansions. I use a somewhat different approach to measure the SNAP benefit level because it is strictly fixed at the federal level in the contiguous U.S. With the lack of opportunity to leverage state policy changes or non-linear changes in benefit amount by family size, I use variation in the *real value* of SNAP benefits that a child’s family is exposed to given the year and market group of residence. Following Bronchetti et al. (2019), the variation in SNAP purchasing power comes from local food prices. I employ a two-way (child and year) fixed effects approach – with a nationally representative sample of 2001 birth cohort – to leverage within child variation in exposure to different levels of SNAP purchasing power and maximum EITC benefits over time among a high intent to treat sample. I demonstrate that changes in the maximum EITC benefits and SNAP purchasing power are independent of one another. As the independence of two measures is one of the key assumptions to identify their plausibly causal interaction effects, I do not use the EITC purchasing power as my main measure; but I show that results are very similar when EITC purchasing power is interacted with SNAP purchasing power. Results indicate evidence of complementary effects between EITC and SNAP on early reading and math scores.

The present study contributes to the public policy, demography, and child development literatures. To my knowledge, this paper is the first to examine the interactive effects of multiple income support programs on child development. Although there is a broader safety net that includes programs beyond EITC and SNAP, these two programs serve as a useful case study of how multiple programs interact to shape marginalized children's development. Moreover, given their importance as key components of the social safety net for low-income working families with children, whether and how they interact to shape child development are important real-world policy questions.

## Background

### Summary of the Program: Earned Income Tax Credit

Because the EITC is “refundable,” the Internal Revenue Service (IRS) refunds the portion of the credit that exceeds a taxpayer’s tax liability, including for low-wage workers with no income tax liability (Marr et al., 2015). The EITC follows a trapezoidal structure as a function of earned income, containing a phase-in region (where the credit increases at the “credit rate”), a plateau region (i.e., the maximum EITC), and a phase-out region. Parameters of the EITC further vary by the number of children in the household and marital status. For instance, the credit rate and the maximum EITC benefit are higher among families with more children. The earning limit also varies; in 2023, depending on the number of children and filing status (single vs. joint), the adjusted gross income limit at which the EITC was completely phased out varied from $46,560 to $63,398 (Crandall-Hollick et al., 2023).

Since its enactment in 1975, the federal EITC has expanded several times, including in 1986, 1990, 1993, and 2009. In addition, from 1986, states started to implement and expand (sometimes removed, and reduced) their own EITC programs. As of tax year 2023, 31 states plus D.C. implemented state EITCs. States also vary significantly in generosity, with state credit rates – typically expressed as a percentage of the federal EITC – differing substantially. Also, some states offer refundable credits, while others offer non-refundable ones. During the study period, spanning tax years from 2003 to 2006, seven states changed their state EITC generosity by adopting a state EITC, expanding their state credit rate, or shifting to a refundable state EITC from a non-refundable one (see Table S1 for details).

### Summary of the Program: Supplemental Nutrition Assistance Program

SNAP was first piloted in 1961 and became a nationwide program in 1974. Eligibility for SNAP is typically determined by the following three tests: (1) gross income test (monthly gross income should be below 130% of the federal poverty line (FPL), e.g., $39,000 for a family of four in 2023)^2^; (2) net income test (gross income minus deductions (e.g., related to dependent care, earned income, child support, medical and excess shelter expenses) should be below 100% of FPL); and (3) asset test (households’ assets should be below a certain value). While SNAP has certain work requirements, as exemptions are often allowed (e.g., for caregivers of children under age six), the program serves both working and non-working individuals. Unlike other programs whose benefit levels vary across states (such as the EITC), the SNAP benefit formula is strictly fixed at the federal level. SNAP benefits are calculated by subtracting 30% of a household’s net income from the maximum SNAP benefit. In the contiguous U.S., the maximum benefit only varies by household size (USDA, 2022).

### The Effect of Each Program on Child Development

Before discussing their potential interactive effects, it is vital to understand how each program may influence children’s early cognitive development. Theoretically, the effect of the EITC on child development in early childhood or early school years is ambiguous, whereas the effect of SNAP is less so. On the one hand, the parental investment perspective and the family stress perspective support the “income pathway” as one potential mechanism through which the EITC and SNAP may improve child development. The parental investment perspective suggests that additional resources from EITC and SNAP would increase the level of parental investment in the child in the form of money and time, such as engaging in enriching activities (e.g., reading) with the child, purchasing cognitively stimulating materials and activities for the child (e.g., books, toys, lessons), and providing more nutritious food (Becker, 1991; Yeung et al., 2002). The family stress perspective suggests that greater income from EITC and SNAP would reduce parents’ stress, which may result in more supportive parenting and better quality of parent–child interactions (Conger et al., 1994; Masarik et al., 2017).

At the same time, EITC incentivizes work (Adireksombat, 2010; Chetty et al., 2013; Eissa et al., 1996). Based on the developmental literature that found maternal labor supply can negatively affect child development during the first few years of a child’s life (Baum, 2003; Waldfogel et al., 2002), the labor supply effect of EITC may mitigate its positive effect on child development in early childhood or kindergarten-entry. This would particularly be the case if increased maternal employment due to EITC reduces time spent with children and/or induces children to attend low-quality childcare among low-income working families, although such evidence is not prominent among existing studies on EITC (Bastian & Lochner, 2022; Bastian & Michelmore, 2018; Michelmore & Pilkauskas, 2021; Morrissey, 2023). Taken together, the EITC’s impact on child development in the early years remains an open empirical question. Regarding SNAP, however, prior research has shown mixed findings (Bitler et al., 2021; Farkhad & Meyerhoefer, 2018), making it less likely that the pathway through which SNAP affects child development operates via labor supply changes.

Little is yet known about how EITC and SNAP each influence early cognitive development among working poor families. Hong and Henly (2020) demonstrate positive relationships between SNAP participation status and early math skills, although their study is not directly comparable to the present study because I focus on the effect of a marginal increase in benefit values. Several research examined other well-being outcomes among older, school-age children. They generally found positive effects of each program on reading and math test scores (EITC: Dahl et al., 2012; Bastian et al., 2018; Chetty et al., 2021; Maxfield, 2015; SNAP: Frongillo et al., 2006; Gassman-Pines et al., 2018), child behavior (EITC: Hamad et al., 2016), education attainment (EITC: Bastian et al., 2018; Maxfield, 2015), and adult health and economic outcomes (SNAP: Hoynes et al., 2016). However, evidence is mixed in a few other studies (e.g., Hamad et al., 2018), and most of the existing research have not focused on the cognitive outcome of young children in or before kindergarten – the research gap that this paper aims to address.

### How would EITC and SNAP Interact to Affect Child Development?

I now turn to the interaction piece – i.e., whether each program’s impact on child development would depend on the other program’s generosity. There can be three possibilities in their interaction effect: positive interaction or complementary effect (i.e., the effect of receiving larger EITC on child development *increases* with larger SNAP, and vice versa); no interaction (i.e., SNAP and EITC *independently* affect child development); negative interaction or substitution effect (i.e., the effect of receiving larger EITC on child development *decreases* with larger SNAP, and vice versa). For simplicity, in this section, I provided examples that illustrate how the effect of EITC may change as the level of SNAP benefits increases; but a similar logic applies to the other case in which the effect of SNAP changes as EITC benefits increase.

**Positive interaction**. Because the EITC is a lump-sum cash benefit provided during tax time, while the SNAP is a monthly in-kind benefit, families may allocate both benefits across different categories. This raises the possibility of mental accounting (Thaler, 1999), which posits that households treat different sources of income differently. Under such circumstances, we may observe positive interaction effects between two programs.

Previous studies demonstrate that parents often consider the EITC refund as “the kids’ money” or “family money” since their children had made them eligible for the credit (Mendenhall et al., 2012; Sykes et al., 2015). Thus, the EITC benefit is often used to meet their child’s “needs” and “wants”, such as clothing, school supplies, toys, and furniture (e.g., cartoon character table and chair) (Mendenhall et al., 2012; Sykes et al., 2015). Also, as it is allocated once a year during tax time, EITC is viewed differently from regular paycheck (Romich et al., 2000) and parents tend to earmark the EITC refund for investment purposes at the expense of other pressing financial needs (Sykes et al., 2015). A body of literature shows that recipients consider the refund as a chance to purchase durable goods or make large purchases (e.g., purchase used cars, repair cars, purchase time saving home appliances or furniture like washing machines and dish washers), to save and invest in education (e.g., school tuition), and to make home improvements or even purchases. Paying off bills and debts is another common usage of the EITC (Goodman-Bacon, & McGranahan, 2008; Jones et al., 2018; Mammen et al., 2006; Mendenhall et al., 2012; Romich et al., 2000; Sykes et al., 2015).

In contrast, SNAP, as an in-kind food benefit, is more restricted in its use and thus may have direct impacts on child nutrition or health. However, as it is considered near-cash, SNAP could still increase inframarginal families’ consumption of non-food basic needs, although possibly to a lesser extent than their spending on food, since a marginal propensity to consume food out of SNAP benefit tends to be higher than it is out of cash income (Beatty et al., 2015; Hastings et al., 2018). Studies have found that SNAP increases spending on essential needs, such as paying rent, utilities, medical costs, and tuition (Kim, 2016; Shaefer et al., 2013), and reduces household debt (Kim et al., 2016).

Thus, if mental accounting is at play, SNAP and EITC may benefit different domains of child development, and in turn, positively interact. For example, if SNAP improves nutrition, children with better nutrition or health may derive greater returns from parental investments in creating enriching home environments (e.g., purchase of educational toys, books) using EITC refunds or EITC-induced earnings (both referred to as “EITC income”). In such cases, larger SNAP benefits could enable the EITC to more effectively improve early cognitive outcomes.

Another mechanism that may lead to positive interaction effects includes spending patterns, that is, how much and in what way parents would spend the EITC income for their child by different levels of SNAP benefits. For instance, if greater SNAP benefits reduce a household’s debt burden by further reducing their out-of-pocket food spending and increasing their ability to pay for other essential needs, this may “free up” a greater portion of EITC income that parents can use for their children. As a result, as the parental investment perspective suggests, parents may purchase more educational materials that are beneficial for children’s learning, compared to otherwise similar families with smaller SNAP benefits who might have to use that portion of EITC for other purposes, e.g., to pay off debt and bills. Similarly, with larger SNAP benefits, parents may allocate a greater portion of their EITC income toward purchasing durables, such as cars and time saving home appliances, which are also common uses of the EITC as described above (Goodman-Bacon, & McGranahan, 2008). These purchases may indirectly improve child development by allowing parents to save time and spend more enriching time with their child, such as reading books together (Morrissey, 2023).

The positive interaction effects may also work through the family stress pathway. Empirical research supports the positive effects of EITC and SNAP (individually) on mental health outcomes, including reductions in stress and depressive symptoms (Boyd-Swan et al., 2016; Schmidt et al., 2023). If stress is a nonlinear function of income, EITC benefits could lead to a larger reduction in parental stress level when families receive larger SNAP benefits, and vice versa, allowing child development to improve more effectively.

**No interaction or negative interaction**. On the other hand, alternative scenarios are possible. For instance, if families view SNAP and EITC as fungible, positive interaction effects would not be expected. Instead, each program would independently influence child development—or, if cash has decreasing marginal utility, negative interaction effects (i.e., substitution effects) could emerge. Moreover, even if mental accounting occurs, whether and how EITC and SNAP positively interact to shape child development remains an empirical question. For example, there will be no interaction if higher SNAP benefits do not lead parents to allocate EITC income in ways that directly or indirectly enhance children’s well-being, such as spending EITC benefits on household appliances that are unrelated to child development. Alternatively, if larger SNAP benefits prompt parents to devote less EITC income or time to children – e.g., when freed-up EITC funds are spent socializing with friends – EITC and SNAP could negatively interact.

### Literature Review: Interactions of Multiple Programs on Child and Family Wellbeing

The current study makes a novel contribution to an emerging, relatively scarce, literature on how exposure to multiple programs interacts to affect child or family well-being. A few existing studies found long-run dynamic complementary effects between multiple educational investments (Ansari et al., 2018; Johnson et al., 2019). For example, Johnson and Jackson (2019) found that exposure to a typical Head Start center in early childhood has greater effects on improving education attainment and wages of poor children and reducing their likelihood of being incarcerated and poor as an adult, when the Head Start exposure is followed by greater K-12 spending, and vice versa. On the contrary, such complementary effects were not found between other types of programs – for instance, Carter (2020) found no clear evidence of complementary effects between preschool age exposure to Head Start and childhood exposure to SNAP on labor market and health outcomes in adulthood. A few other studies evaluated how the EITC and a labor regulation policy (i.e., minimum wage) interact. Neumark and Wascher (2011) demonstrate that a higher minimum wage increases the positive impact of the EITC on employment and earnings of single women with children, while a higher minimum wage exacerbates the adverse effect of EITC on employment and earnings of people who are ineligible for the EITC: less-skilled minority men without children. Lenhart and Chakraborty (2024) show that a higher minimum wage reduces suicide and assault rates only in states with EITC policies, also indicating complementary effects between them.

Several studies also considered the impacts of aggregated benefits from more than two safety net programs, while they did not directly test their interaction effects. Schmidt et al. (2023) found that a combined value of cash and food benefit programs reduces severe psychological distress, after accounting for the fact that programs can influence each other's eligibility and benefit amounts. Similarly, Reynolds and Homan (2023) reported that a combined value of EITC, SNAP, minimum wage, and unemployment insurance is associated with reductions in preterm births and low birthweight births. The present study extends this emerging literature by examining whether and how multiple income support programs interact to influence children’s early cognitive development.

## Data and Methods

Data comes from the Early Childhood Longitudinal Study-Birth Cohort (ECLS-B), which follows a nationally representative cohort of over 10,000 children born in 2001 from birth through kindergarten entry when they were 9 months old (wave 1; 2001–2002), 2 years old (wave 2; 2003–2004), 4 years old (wave 3; 2005–2006), kindergarten-entry age (wave 4; 2006–2007), and kindergarten-entry age (wave 5; 2007–2008). Within each wave, children were interviewed at various time points. For instance, assessment months vary between August, 2005-June, 2006 in wave 3, September, 2006 -March, 2007 in wave 4, and November, 2007-March, 2008 in wave 5. In wave 5, specific groups of children were only invited to take the survey, which included children who did not enter kindergarten in wave 4, children who were repeating kindergarten in this wave, and twins of these children (Snow et al., 2009). The ECLS-B recommends combining wave 4 and wave 5 to create a kindergarten-entry wave (“wave k”) that is nationally representative of the 2001 birth cohort at their kindergarten entry period (Najarian et al., 2010; Snow et al., 2009). Following this recommendation, I constructed wave k by including data from the wave in which each child first entered kindergarten (i.e., wave 4 or wave 5). The ECLS-B is particularly suitable for this study since it contains a rich set of early childhood family and child characteristics reported by parent respondents, state of residence, county of birth, and development outcomes. To my knowledge, there are no nationally representative survey datasets of a newer birth cohort that provide similar advantages (see Online Supplement Section A). Considering that 96–99% of survey respondents were mothers across all waves, I hereafter refer to parent respondents as mothers.

The study sample comprises children from wave 3 and wave k, because early reading and math outcomes were not measured in wave 1 and wave 2. The study’s population of interest is families who participate in both EITC and SNAP. However, given that only SNAP receipt status is available from the ECLS-B, the main study sample includes children living in households where at least one parent was employed and who reported receiving SNAP benefits in both survey waves. Specifically, in wave 3, respondents were asked whether they had received SNAP “since the child turned age 2,” and in wave k, whether they had received SNAP “since the child turned age 4 or 5,” depending on whether it was wave 4 or 5. This is a high intent-to-treat sample that comprises families who are likely to receive both SNAP and EITC. Because work status could be affected by one or both of the programs, I used their work status measured in wave 2. While the advantage of using this sample is clear, one might be concerned about a selection effect of SNAP purchasing power on SNAP participation, which may – if exists – bias my estimate. As indicated in Table S2, SNAP purchasing power does not lead to changes in SNAP participation among likely SNAP-eligible households, showing that there is no evidence of selection into SNAP receipt. Furthermore, recognizing the potential underreporting issue with the self-reported SNAP receipt variable, I considered a broader sample of unmarried mothers with high school degree or below in sensitivity analyses.

Among working SNAP households (N = 1650 household-wave observations), my main study sample is further limited to households living in contiguous U.S. states (excluding Alaska and Hawaii) and those with birth county information (N = 1550). This restriction is necessary because the SNAP purchasing power data are merged with the ECLS-B data based on county of birth and cannot be estimated in Alaska and Hawaii using existing information. Then, I limited to households who have non-missing information on dependent variables (N = 1450–1500) and covariates (N = 1400), and to those who are observed in both wave 3 and wave k, resulting in a sample of 1300 household-wave observations (650 households or children). In conforming to NCES guidelines for the ECLS-B, the sample sizes were rounded to the nearest 50.

### Measures

**Child development outcomes** In wave 3 to wave k, early reading and early math scores were directly assessed through one-on-one tasks conducted by the home interviewer. The early reading assessment tool was developed based on validated, standardized instruments such as the Preschool Language Assessment Scale (PreLAS) 2000, Peabody Picture Vocabulary Test (PPVT), and Preschool Comprehensive Test of Phonological and Print Processing (PreCTOPPP). Early reading skills measure both language and literacy skills, such as English language skills, word recognition, letter knowledge, letter-sound knowledge, vocabulary, and developing interpretation. The early math assessment tool, also developed from validated, standardized instruments such as the Test of Early Mathematics Ability-3, tests knowledge of number sense, operations, measurement, data analysis, patterns, and geometry (Snow et al., 2009). Also, some of the items in early reading and early math assessments were taken from the Early Childhood Longitudinal Study, Kindergarten Class of 1998–99 (ECLS-K) (Najarian et al., 2010). Overall longitudinal scale scores for early reading and early math were calculated using Item Response Theory (IRT) procedures. The IRT scale scores represent estimates of the number of correct answers that would have been expected if a child had answered all items in each assessment. The reliability of the IRT based early reading and math scores was tested and was proven to be high enough (Najarian et al., 2010).

**SNAP purchasing power** I calculated SNAP purchasing power as the ratio of the maximum SNAP benefit (which does not vary in the contiguous U.S.) to the regional cost of the Thrifty Food Plan (TFP), following Bronchetti et al. (2019). This measure was merged with the ECLS-B data based on the market group of residence (using a child’s birth county) and assessment year. TFP is the least expensive nutrition plan, established by the USDA, that contains recommended amounts of foods in 29 food categories for a “reference” family, defined as a family of four comprised of an adult male and female (age 20–50) and two children (age 6–8 and age 9–11). Since the local TFP cost represents food costs for a family of four, I also used the maximum SNAP benefit for a family of four. The TFP price is particularly relevant to the study’s context, since the maximum SNAP benefits are legislated based on the national average cost of the TFP and food prices tend to be correlated with the prices of other goods or services (Bronchetti et al., 2019).

The regional TFP price was estimated using the Quarterly Food-at-Home Price Database (QFAHPD). The QFAHPD, constructed by USDA Economic Research Service researchers using Nielsen Homescan data, has quarterly prices for 52 food-at-home categories (e.g., 12 vegetables groups, 3 fruit groups, 6 dairy groups) for each of the 35 market groups from 1999 to 2010. 35 market groups are exhaustive of the contiguous U.S. and each market group comprises a set of counties. A market group includes one metropolitan area, e.g., Boston, Chicago, San Francisco, Los Angeles, when there are no sample size concerns, while in otherwise instance, a few metropolitan areas are aggregated into one market group (e.g., Indianapolis, Detroit, Milwaukee, and Grand Rapids constitute “Metro Midwest 1”). For nonmetro areas, they are aggregated based on 9 census divisions. Thus, this resulted in 26 market groups for metropolitan areas, and 9 market groups for nonmetropolitan areas (Todd et al., 2010). Figure S2 shows a map of 35 market groups, reprinted from Todd and colleagues (2010). I also provided a more detailed description of how yearly TFP prices are estimated at the market group level in Online Supplement (see Section A).

**Fig. 1:**
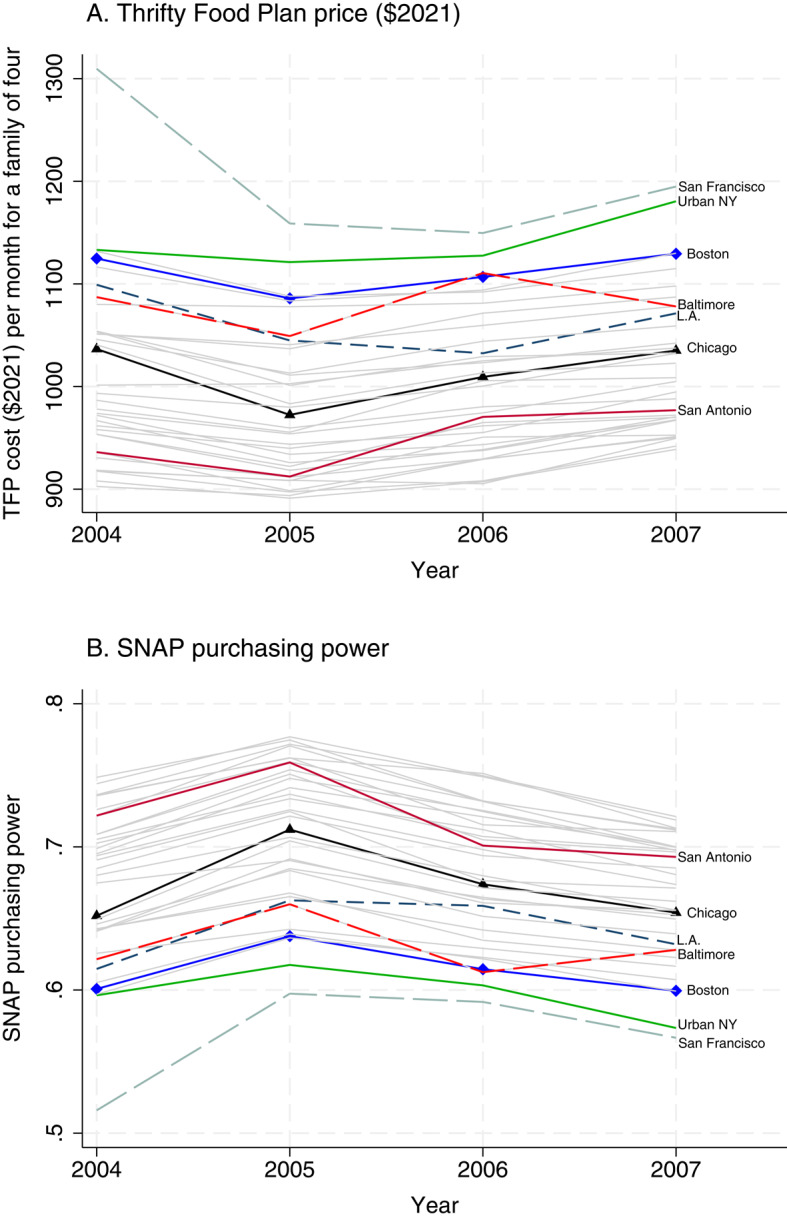
Thrifty Food Plan Price and SNAP Purchasing Power by Market Groups from 2004 to 2007 *Notes*: In panel A and panel B, each line indicates a market group’s trend in Thrifty Food Plan price and SNAP purchasing power, respectively. The SNAP purchasing power data are from Bronchetti et al. (2019)

Figure 1 shows the trends in TFP price and SNAP purchasing power across time by each market group, with relatively large cities highlighted as colored lines. According to Fig. 1, there are large variations in TFP price (panel A) and therefore, SNAP purchasing power (panel B), not only across market groups but also over time within each market group.

**Fig. 2:**
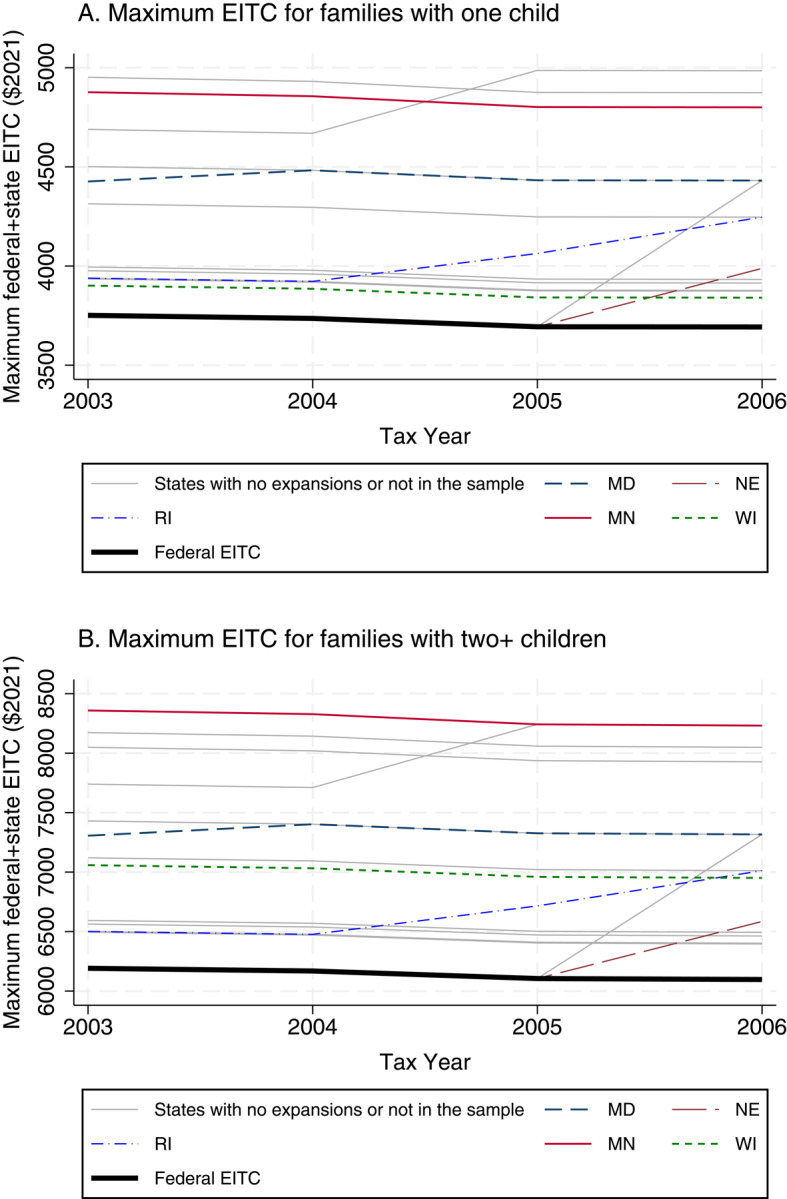
Maximum State and Federal EITC Benefits from Tax Year 2003 to 2006 Notes: In figures in panel A and panel B, states that implemented the state EITC or changed the state credit rate during tax years 2003–2006 and are represented in the study sample are indicated as colored lines. States that have different state credit rates by the number of children in the household are also indicated as colored lines. The following states that expanded state EITCs but are not included in the study sample (DC, DE, VA) or have their own state EITC but did not experience changes in state EITC credit rate during the study period are indicated as grey lines (IA, IN, IL, KS, MA, ME, NJ, NY, OK OR, VT). States without their own state EITC programs, who are assigned with the maximum federal EITC benefit, are indicated as the black thick line. Data come from National Bureau of Economic Research (2019) and Komro et al. (2020)

**Maximum EITC benefits** Drawing on multiple data sources, including NBER TAXSIM data (National Bureau of Economic Research [NBER], 2019) and the EITC data created by Komro et al. (2020), I computed the maximum federal and state EITC benefits by multiplying the maximum federal EITC benefit by (1 + state credit rate).^3^ This maximum EITC variable was merged with the ECLS-B data by state of residence,^4^ survey year, and the number of children living in household in the relevant tax year. During the study period, state EITC policy changes were modest, with only a few states changing EITC credit rates or adopting a state EITC. Figure 2 illustrates the trends in maximum federal and state EITC benefits among states that are represented in the sample and that expanded their EITC during tax years 2003–2006 (i.e., 2004–2007 in terms of receipt), which include Maryland, Nebraska, and Rhone Island. See panel A in Table S1 for a full list of states with EITC expansions during the study period. Therefore, in this study, the majority of the variation in maximum federal and state EITC benefits stems from differences in the number of children. Specifically, the maximum federal EITC benefit increases non-linearly when the number of children rises from one to two or more, and some states (such as Minnesota and Wisconsin) offer higher state credit rates for households with more children (see panel B in Table S1). Prior research also identified the effect of the EITC or Child Tax Credit (CTC) benefits by leveraging within-household changes in the number of children over time (Pilkauskas et al., 2019) or comparing households with different number of children residing in the household (Pac et al., 2024; Pilkauskas et al., 2024).

EITC does not face the same purchasing power problem as SNAP. Although the federal EITC is not adjusted for local costs of living, many states offer their own EITCs. As a result, the variation in EITC purchasing power *across states* is likely smaller than in SNAP (because there are no state-level SNAP benefits that could mute the purchasing power difference), whereas its variation may increase *within a given state* after local costs of living are accounted for. But, as I show below, the EITC purchasing power variable does not fully satisfy one of the model's assumptions. I therefore used the maximum EITC benefit as my primary variable, although I later show in sensitivity analyses that the results are very similar when EITC purchasing power is interacted with SNAP purchasing power.

**State-, county-, and individual-level control variables** I controlled for various state-, county, and individual-level characteristics that may influence child development and may be associated with local food prices and/or maximum federal and state EITC benefits. As state-year level variables, I adjusted for unemployment rate (from Bureau of Labor Statistics (BLS)), poverty rate (from U.S. Census Bureau’s Small Area Income and Poverty Estimates), maximum TANF benefit for a family of four (from University of Kentucky Center for Poverty Research), per-capita income (from Bureau of Economic Analysis), state minimum wage (from BLS and Tax Policy Center), and upper income eligibility limit of Medicaid/SCHIP for children (from Kaiser Family Foundation and National Governor’s Association). Moreover, the prices of other goods were included: the U.S. Department of Housing and Urban Development’s (HUD’s) Fair Market Rent (FMR) for 2-bedroom units, which estimate 40th percentile gross rents for standard quality units by county and year (from HUD Office of Policy Development and Research), and regional Consumer Price Index (CPI) for apparel, transportation, education, and recreation (from U.S. Census Bureau and BLS). During the study period, regional CPIs are available for each year in 27 metro core based statistical areas, while in the rest of the regions, they are available in 11 sampling units that consist of different census regions by population sizes (< 50,000; 50,000–1.5 million; > 1.5million). The correlations between TFP price and other local prices are shown in Table S3. These additional price controls, along with state unemployment and poverty rates, help ensure that the SNAP purchasing power variable captures the role of SNAP purchasing power itself, rather than the broader effects of overall local prices or living in different labor markets.

As individual-year level characteristics, I adjusted for child age (in months) and mother’s age (in years) at the survey assessment and their squared terms, parent’s highest education attainment (below high school, high school degree, some college, BA or higher), household size (two, three, four, five, six or more), mother’s marital status (not married, married), and household’s Medicaid receipt status and urbanicity (rural, urban, urban cluster).

Table 1 shows descriptive statistics of all study variables by survey waves in the study sample. The negative values in the standardized child outcomes demonstrate that children in the sample had lower scores than the national average, represented by the ECLS-B children. Furthermore, 63% of children had parents with a high school degree or less, 41% had married mothers, and 89% received Medicaid (see “Total” column). The majority of the sample lived in families of four or more people (78%) and with two or more children (85%) and lived in urban or urban-cluster areas (77%). Also, although not shown in Table 1, White children constitute the largest share of the sample (38%), followed by Black (33%), Hispanic (21%), and other race/ethnicity (7%). Several variables show statistically significant changes from wave 3 to wave k. As expected, SNAP purchasing power, maximum EITC benefits, and most of the state- and county-level characteristics changed statistically significantly over time. There were relatively few changes among child and family covariates and outcomes; those that showed changes include Medicaid receipt status (marginally significant), number of children, child and mother’s age, and early math skills.^5^

**Table 1:** Sample Characteristics of the Sample by Study Waves

	Mean(SD) / Percent			
	Wave 3	Wave K	Total	Sig
Std. early reading	− 0.42	− 0.36	− 0.39	
	(0.80)	(0.89)	(0.84)	
Std. early math	− 0.49	− 0.38	− 0.44	*
	(0.86)	(0.90)	(0.88)	
SNAP purchasing power	0.70	0.72	0.71	***
	(0.05)	(0.04)	(0.05)	
Maximum federal and state EITC benefit ($2021)	5742.50	5915.77	5829.14	***
	(1272.40)	(1118.07)	(1200.40)	
Household size (%) Two (Ref.)				
Three	16.75	14.01	15.38	
Four	25.96	25.65	25.81	
Five	24.81	25.67	25.24	
Six or more	26.34	28.44	27.39	
Two or more children (vs. one child) (%)	82.62	87.26	84.94	**
Married (vs. Not married) (%)	41.52	41.38	41.45	
Urbanicity (%) Rural (Ref.)				
Urban	62.6	61.74	62.17	
Urban-cluster	14.84	14.82	14.83	
Received Medicaid (vs. did not receive) (%)	90.14	86.88	88.51	†
Parent's highest education attainment (%) No high school degree (Ref.)				
High school degree	44.89	40.8	42.84	
Some college	32.03	33.4	32.71	
BA or higher	3.73	5.14	4.43	
Child age at the interview (in months)	52.10	68.21	60.16	***
	(3.79)	(4.48)	(9.07)	
Mother's age (in years)	28.90	30.13	29.52	***
	(6.22)	(6.28)	(6.28)	
Unemployment rate	5.57	5.09	5.33	***
	(0.86)	(1.07)	(1.00)	
Percent of poverty	13.49	14.10	13.80	***
	(2.77)	(2.91)	(2.86)	
Per-capita income ($2021 in thousands)	46.64	47.78	47.21	***
	(6.37)	(6.54)	(6.48)	
Maximum TANF benefits for a family of four ($2021 in hundreds)	6.04	5.83	5.93	***
	(2.57)	(2.52)	(2.54)	
Medicaid income eligibility limit as % of FPL	2.22	2.22	2.22	†
	(0.44)	(0.44)	(0.44)	
Minimum wage ($2021)	7.61	7.64	7.63	
	(0.81)	(1.00)	(0.91)	
Fair market rent for 2-bedroom ($2021)	955.15	939.67	947.41	*
	(313.77)	(274.99)	(295.01)	
Regional CPI for apparel costs	106.06	105.42	105.74	*
	(16.95)	(18.61)	(17.79)	
Regional CPI for education costs	110.41	113.25	111.83	***
	(4.02)	(4.89)	(4.70)	
Regional CPI for recreation costs	108.78	110.06	109.42	***
	(3.93)	(4.65)	(4.35)	
Regional CPI for transportation costs	141.69	153.12	147.41	***
	(26.91)	(28.53)	(28.30)	

All estimates are weighted, except for sample sizes. 'Std.' stands for standardized. 'Sig.' column shows whether a given variable is statistically significantly different between wave 3 and wave k. † p < 0.1, * p < 0.05, ** p < 0.01, *** p < 0.001. Data come from wave 3 and wave k in the ECLS-B (N = 1300)

### Analytic Strategy

Using a two-way (child and year) fixed-effects model, I derived an intent to treat estimate of the interaction effect between two continuous treatment variables – i.e., maximum EITC benefits and SNAP purchasing power. I analyzed the following specification.

$$\begin{gathered} Y_{{itsmc}} = \alpha _{0} + \delta _{1} {\mathrm{maxEITC}}_{{is,t - 1}} + \delta _{2} \frac{{{\mathrm{maxSNAP}}}}{{{\text{TFP price}}}}_{{m,t - 1}} + \delta _{3} {\mathrm{maxEITC}}_{{is,t - 1}} \hfill \\ \times \frac{{{\mathrm{maxSNAP}}}}{{{\text{TFP price}}}}_{{m,t - 1}} + X_{{it}}' \beta + \phi _{{s,t - 1}}' \mu _{1} + \theta _{{c,t - 1}}' \mu _{3} + \lambda _{t} + \gamma _{i} + \varepsilon _{{itsmc}} \hfill \\ \end{gathered} $$

where *i* indicates child, *t* indicates wave 3 and wave k, *s* indicates state of residence, *m* indicates market group of residence, and *c* indicates county of residence. To ensure that SNAP purchasing power and maximum EITC benefits indicate the generosity of benefits that families received before their survey year, I lagged those measures by one year. In terms of tax year, this is equivalent to lagging maximum EITC benefits by two years, because households would not receive their EITC benefits for the current tax year until the following year. In order to obtain a meaningful and realistic interpretation of the main effect of each measure, I mean centered SNAP purchasing power (centered at 0.71; 1-unit = 0.1) and maximum EITC benefits (centered at $5830, 1-unit = $1000 in 2021 dollars).

The child fixed effects model exploits changes within a child or family over time. Child fixed effects, ${\gamma }_{i}$, control for all stable child or family characteristics that may confound the relationship between the SNAP and EITC measures and child outcomes, such as child innate cognitive ability, permanent disability, child temperament, and parental fixed approach to early child learning (i.e., parenting). I did not include state or market group fixed effects, as they are subsumed by child fixed effects. Year (or survey wave) fixed effects, ${\lambda }_{t}$, account for time-varying confounders that affect all children in the same survey wave (e.g., macro-economic shocks or federal-level policy changes). The remaining confounders are therefore time-varying factors that vary across individuals. To account for this, as described earlier, I adjusted for a vector of state level characteristics, ${\phi }_{s,t-1}$, local prices of other goods, ${\theta }_{c,t-1}$, and child and family characteristics, ${X}_{it}$, that change over time.

$${\delta }_{1}$$ indicates the main effect of a $1000 increase in the maximum EITC at the mean of SNAP purchasing power, while $${\delta }_{2}$$ indicates the main effect of a 0.1 increase in SNAP purchasing power at the mean of maximum EITC benefits. The key coefficient of interest, $${\delta }_{3}$$, indicates whether the association between a $1000 increase in the maximum EITC benefits and child outcomes varies by different levels of SNAP purchasing power, and vice versa. This is identified because some children experienced increases in maximum EITC benefits when SNAP purchasing power increased, while other children experienced similar changes in maximum EITC benefits when SNAP purchasing power decreased, and vice versa. A positive value of $${\delta }_{3}$$ would indicate that there is a positive interaction effect. Combining the main effect of each program with $${\delta }_{3}$$, I computed the marginal effect of maximum EITC benefits (or SNAP purchasing power) at varying levels of SNAP purchasing power (or maximum EITC benefits).

The variation in SNAP purchasing power within each child comes from variation remaining in the TFP price over time within a market group of residence, after parsing out the effects of state economic and labor market conditions and safety net policies, and local prices of other goods. Though it is difficult to accurately explain what is driving the remaining variation in local TFP cost, it may be driven by supply chain dynamics (e.g., which grocery chains are in a market, which wholesalers and retailers are in a market), local agricultural production, local supply shocks, and demand conditions (Bronchetti et al., 2019). Figure S1 (in Online Supplement) plots residuals from the regressions of TFP cost on covariates included in my main specification and they illustrate a fair amount of idiosyncratic variation.

The variation in maximum EITC benefits within each child comes from (i) changes in the number of children across the two relevant tax years (from one child to two or more children); and (ii) changes in state EITC policies over time within a state of residence. But as noted earlier, additional births are largely driving the variation in maximum EITC benefits. The key assumption underlying this approach is that households experiencing changes in the number of children from one to two or more do not differ from other households (i.e., those without additional births or with increases from two to three or more children) in ways that affect the focal child’s early cognitive development. To investigate the plausibility of this assumption, I compared descriptive statistics of demographic and socioeconomic variables between the two groups. I found no evidence of statistically significant (p < 0.05) differences in all variables (see Table S4), which supports the validity of using the variation based on additional births. Although I cannot rule out the possibility of their differences in unobservable characteristics, the falsification test presented in a later section alleviates this concern. In Table S4, I also show that when households whose number of children changes between one and two or more are excluded from the sample, the main effect of SNAP purchasing power and, importantly, the interaction effect remain substantively similar, indicating that the interpretation of overall findings is unchanged. The main effect of maximum EITC benefits becomes smaller and less precise, which is expected, because their variation is reduced after those households are removed.

Both SNAP and EITC measures may also show variation due to families moving across states or market groups. However, as noted in footnote 4, my findings are not sensitive to excluding families who moved across states, who represent only 7% of the sample. In addition, because children were assessed in different years within a given survey wave, their variations may also result from differences in assessment timing, which are likely idiosyncratic (see Online Supplement Section A).

We can interpret the main effect and the interaction effect as causal to the extent that (i) changes in SNAP purchasing power and maximum EITC benefits are exogenous – i.e., not driven by correlates of changes in child outcomes – and that (ii) those changes in SNAP purchasing power and maximum EITC benefits are independent of one another, after adjusting for all covariates (Almond et al., 2013). Although the first assumption is not testable, to assess its plausibility, I performed a falsification test and a few robustness checks, along with the tests conducted above. The second assumption is formally tested, which is presented below. To adjust for survey nonresponse and disproportionate sampling, and to allow for making inferences about the national population, the survey weights were applied to all analyses. Two-way clustering method was used to cluster standard errors at the market group- and the state-level, but results were robust to clustering standard errors at one of those levels.

**Testing the independence of maximum EITC benefits and SNAP purchasing power** In this subsection, I tested the second model assumption, i.e., the independence of SNAP purchasing power and maximum EITC benefit, by regressing the SNAP purchasing power on the maximum EITC and the other way around. As the maximum EITC benefit, which is based on tax years, is measured prior to the SNAP purchasing power and is not a function of earnings, I can rule out the possibility of higher SNAP purchasing power (i.e., lower food prices) being associated with lower wages and in turn with the maximum EITC benefit. Moreover, because the variation driving changes in the SNAP purchasing power and the maximum EITC benefit are at different levels, they are likely independent of each other. Indeed, Table 2 indicates that the coefficients are very small and statistically nonsignificant in either way, formally showing that changes in SNAP purchasing power are independent from those in maximum EITC benefits. On the other hand, the purchasing power of maximum EITC benefits (i.e., calculated as the ratio of the maximum federal and state EITC benefits to the market group level TFP prices) is correlated, although marginally significantly, with the SNAP purchasing power (available upon request).

**Table 2:** Independence of the Maximum EITC Benefits and SNAP Purchasing Power

	SNAP purchasing power	Maximum EITC
Maximum EITC	− 0.00	
	(0.00)	
SNAP purchasing power		− 0.17
		(2.51)

All estimates are weighted, except for sample sizes. All covariates are controlled, including child and year fixed effects, state-level economic and policy characteristics, local prices of other goods, and child or family level characteristics (although results do not change when the state-level covariates, other prices, and individual characteristics are not controlled; available upon request). † p < 0.1, * p < 0.05, ** p < 0.01, *** p < 0.001. Data come from wave 3 and wave k in the ECLS-B (N = 1300)

## Results

Table 3 presents the main findings. The main effect of maximum EITC benefits ranges from 0.04 SD to 0.07 SD, while that of SNAP purchasing power ranges from 0.17 SD to 0.22 SD across early reading and early math outcomes. However, both effects are statistically nonsignificant due to large standard errors. These findings hold in a model that includes only their main effects and excludes the interaction term (see Table S6).

**Table 3:** Interaction Effects Between the Maximum EITC and the SNAP Purchasing Power

	Reading	Math
*Regression coefficients*		
Maximum EITC (centered)	0.07	0.04
	(0.06)	(0.06)
SNAP purchasing power (centered)	0.17	0.22
	(0.18)	(0.18)
Maximum EITC $$\times $$ SNAP purchasing power	0.18**	0.21*
	(0.05)	(0.08)
Mean of outcome	− 0.39	− 0.43
*Marginal effects of maximum EITC by SNAP purchasing power levels*		
With 10% decrease	− 0.05	− 0.11
	(0.06)	(0.08)
Average	0.07	0.04
	(0.06)	(0.06)
With 10% increase	0.20*	0.19*
	(0.09)	(0.08)
*Marginal effects of SNAP purchasing power by maximum EITC benefit levels*		
With $1000 decrease	− 0.01	0.01
	(0.20)	(0.21)
Average	0.17	0.22
	(0.18)	(0.18)
With $1000 increase	0.35*	0.44*
	(0.18)	(0.19)

All estimates are weighted, except for sample sizes. Only the key coefficients are displayed in the table, but all covariates are controlled, including child and year fixed effects, state-level economic and policy characteristics, local prices of other goods, and child or family level characteristics. Table S12 in Online Supplement shows the full regression results. † p < 0.1, * p < 0.05, ** p < 0.01, *** p < 0.001. Data come from wave 3 and wave k in the ECLS-B (N = 1300)

On the other hand, the coefficients on the interaction terms are statistically significant and positive for both outcomes, suggesting that EITC and SNAP positively interact to influence early cognitive development. These results underscore the importance of testing for interaction effects: without the interaction term, one might incorrectly conclude that SNAP purchasing power and maximum EITC benefits are not associated with early cognitive development.

**Fig. 3:**
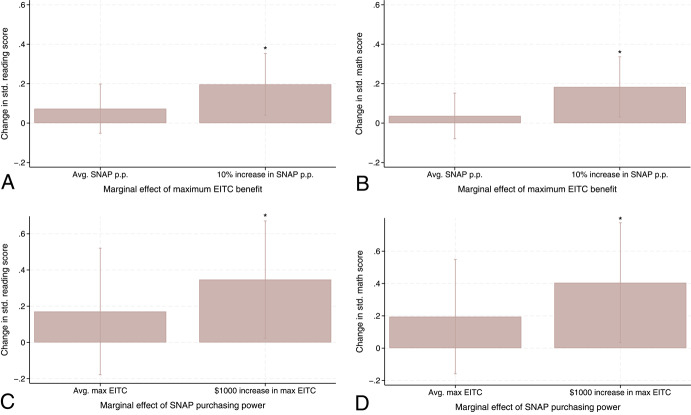
Marginal Effects of Each Program on Cognitive Development by the Other Program’s Generosity *Notes*: The average SNAP purchasing power is 0.71; the average maximum federal and state EITC is $5830. The estimates of all marginal effects and whether marginal effects statistically significantly change as the level of the other program’s generosity increases (i.e., the interaction effect) is indicated in Table 3. Stars above confidence intervals show the statistical significance of the marginal effect of each program. † p < 0.1, * p < 0.05, ** p < 0.01, *** p < 0.001. Data come from wave 3 and wave k in the ECLS-B (N = 1300)

To better show the magnitude of the interaction effect, I present a series of graphs in Fig. 3 that illustrate how the marginal effect of maximum EITC benefits changes as the level of SNAP purchasing power changes (at the baseline mean, 10% increase from the mean, which approximately amounts to an additional $949-$1344 (in 2021 dollars) per year for an average household size in the study sample^6^) and how the marginal effect of SNAP purchasing power changes as the level of maximum EITC benefits changes (at the baseline mean, $1000 increase from the mean). The specific values of those marginal effects are displayed in Table 3. Panel A in Fig. 3 shows that, for children exposed to the mean SNAP purchasing power, a $1000 increase in the maximum EITC benefit led to a statistically nonsignificant 0.07 SD increase in early reading scores (which equals the main effect of EITC in Table 3). However, with a 10% increase in SNAP purchasing power, a $1000 increase in the maximum EITC benefit results in a statistically significant 0.20 SD increase in early reading scores. The same trend appears for early math scores (Panel B). With a 10% increase in SNAP purchasing power from its mean, a $1000 increase in the maximum EITC benefit is linked to a larger improvement in early math scores, with the magnitudes increasing from 0.04 SD (statistically insignificant) to 0.19 SD (statistically significant). I also found a similar result on the marginal effect of SNAP purchasing power: Panel C shows that, with a $1000 increase in maximum EITC benefits from the baseline mean, the marginal effect of SNAP purchasing power on early reading skills becomes statistically significant and rises from 0.17 SD to 0.35 SD. Similarly, for early math skills (see Panel D), the marginal effect of SNAP purchasing power increases from 0.22 SD to 0.44 SD and reaches statistical significance, with a $1000 increase in maximum EITC benefits from the mean.

Taken together, these graphs consistently demonstrate that one program becomes effective at improving early cognitive development among children in preschool to kindergarten-entry years when it is coupled with more generous benefits from the other program.

### Falsification and Robustness Tests

Next, I conducted a falsification test and a series of robustness tests. As a falsification test, I reran the main model for the following placebo sample where I expect to see null or very small effects of both programs: children whose mother is married and college educated, a group with very low rates of SNAP participation. Panel A in Table 4 shows the estimates. Reassuringly, all estimates of the main effects of each measure and their interaction effect are statistically nonsignificant and very small and in the opposite direction from my main results. This confirms that the positive interaction effect does not exist in the placebo sample (nor do the main effects of each program), suggesting that bias due to unobserved confounding factors is minimized through my analytic strategy.

**Table 4:** Falsification and Robustness Test Results

	Reading	Math
Panel A. Placebo group (married, college-educated)		
Maximum EITC (centered)	− 0.05	− 0.04
	(0.04)	(0.04)
SNAP purchasing power (centered)	− 0.15	− 0.07
	(0.15)	(0.11)
Maximum EITC $\times$ SNAP purchasing power	− 0.01	0.02
	(0.05)	(0.04)
N	3850	3850
Panel B. SNAP families above deep poverty (> 50% of FPL)		
Maximum EITC (centered)	0.09†	0.03
	(0.05)	(0.05)
SNAP purchasing power (centered)	0.02	0.02
	(0.18)	(0.13)
Maximum EITC $\times$ SNAP purchasing power	0.26***	0.26***
	(0.06)	(0.06)
N	1350	1350
Panel C. Overall SNAP households		
Maximum EITC (centered)	0.05	0.02
	(0.04)	(0.04)
SNAP purchasing power (centered)	− 0.05	0.04
	(0.19)	(0.15)
Maximum EITC $\times$ SNAP purchasing power	0.12**	0.21***
	(0.04)	(0.06)
N	2050	2050
Panel D. Unmarried mothers with high school degree or below		
Maximum EITC (centered)	− 0.05	0.04
	(0.06)	(0.07)
SNAP purchasing power (centered)	0.04	0.03
	(0.19)	(0.16)
Maximum EITC $\times$ SNAP purchasing power	0.05	0.16**
	(0.05)	(0.05)
N	1250	1250

Each panel is a separate regression. All estimates are weighted, except for sample sizes. Only the key coefficients are displayed, but all covariates are controlled, including child and year fixed effects, state-level economic and policy characteristics, local prices of other goods, and child or family level characteristics. Table S12 in Online Supplement shows the full regression results. Data come from wave 3 and wave k in the ECLS-B. † p < 0.1, * p < 0.05, ** p < 0.01, *** p < 0.001

Second, I analyzed the main model using the following alternative samples to test the robustness of my results: (i) SNAP households above deep poverty, defined as having household income (which does not include public benefits) above 50% of the FPL in wave 2; (ii) overall SNAP households, without conditioning on work or deep poverty status; (iii) unmarried mothers with high school degree or below education. I expect to find qualitatively similar positive interaction effects in the first sample. On the other hand, I expect that the interaction effects will be weaker in the second and third samples compared to my main sample, given that they may comprise non-working households who are not eligible for the EITC or households who are ineligible for both programs. Findings align with these expectations (see Table 4). In panel B, I show that among SNAP households above deep poverty, the interaction coefficients are similar to those from the main model. In panel C, I also found substantively similar, but smaller, interaction effects for SNAP households overall. In panel D, the positive interaction effect held for early math skills among unmarried mothers with high school degree or less, while it became statistically nonsignificant for early reading skills. Regarding main effects, it is notable that the main effect of SNAP purchasing power is smaller in these alternative samples compared to my main sample, while that of maximum EITC benefits is not as sensitive.

Third, I tested the interaction effects between EITC purchasing power and SNAP purchasing power. Table S7 shows that the interaction effects are consistently positive, with similar or slightly larger magnitudes, across four different samples for both early reading and early math skills. Additionally, I investigated whether there is any evidence of SNAP purchasing power reflecting the effect of living in different labor markets, rather than that of SNAP purchasing power itself. Reassuringly, Table S8 shows that SNAP purchasing power is not statistically significantly related to income and income to needs ratio. Lastly, findings are also robust to controlling for families’ participation in other cash-based means-tested programs (SSI, SSDI and TANF) (see Table S9).^7^

### Mechanisms

In this section, I examined the mechanisms through which the positive interaction between the EITC and SNAP may occur. A few possible pathways were described in the Background section, including: the two programs influencing different domains (e.g., nutrition vs. educational inputs); complementary changes in spending on children or on purchases that could indirectly benefit children; and complementary decreases in parental stress.

Because there is no (or very little) time lag between mediators and child outcomes measured in the same wave, it is methodologically challenging to do a formal mediation analysis with a child fixed effects model. Also, there is only a limited set of related mediators in the ECLS-B data – as there is lack of information on households’ spending on children’s educational or non-educational materials (such as educational toys and games, school supplies, clothing, and furniture), transportation, home appliances, etc. Hence, I conducted a preliminary test, analyzing the interaction effects of maximum EITC benefits and SNAP purchasing power on potential mediators related to those pathways, considering mother’s depressive symptoms (binary), the number of books at home (standardized to mean of 0 and SD of 1), cognitive activity index (range: 1–4), daily reading time (in minutes), and healthy eating index (standardized) (see Table S11 in Online Supplement for details about these variables).

Table 5 presents the estimates. First, I did not find statistically significant main effects and interaction effects on maternal depressive symptoms and severe depressive symptoms. Regarding the number of books at home, the main effect of SNAP purchasing power is marginally significant, suggesting that a 0.1 increase in SNAP purchasing power is associated with a 0.24 SD increase in the number of books at home among children exposed to the mean level of maximum EITC benefits. On the other hand, the main effect of maximum EITC benefits and the interaction effect are statistically nonsignificant.

**Table 5:** Mechanisms: Interaction Effects Between Maximum EITC and SNAP Purchasing Power on Potential Mediators

	Severe depressive symptoms	Depressive symptoms	Books (std.)	Cognitive activity	Read time	Healthy eating (std.)
Maximum EITC (centered)	0.02	− 0.02	− 0.04	− 0.04	1.60	0.05
	(0.03)	(0.03)	(0.03)	(0.05)	(1.12)	(0.08)
SNAP purchasing power (centered)	− 0.06	0.05	0.24†	− 0.18	4.55	0.73*
	(0.09)	(0.11)	(0.14)	(0.15)	(4.22)	(0.32)
Maximum EITC $\times$ SNAP purchasing power	0.00	0.03	0.02	− 0.10	2.51*	0.27†
	(0.04)	(0.04)	(0.06)	(0.07)	(1.22)	(0.15)
N	1250	1250	1300	1300	1250	1300
Mean of outcome	0.14	0.31	− 0.30	2.87	25.44	0.07

All estimates are weighted, except for sample sizes. Only the key coefficients are displayed in the table, but all covariates are controlled, including child and year fixed effects, state-level economic and policy characteristics, local prices of other goods, and child or family level characteristics. Table S13 in Online Supplement shows the full regression results. † p < 0.1, * p < 0.05, ** p < 0.01, *** p < 0.001. Data come from wave 3 and wave k in the ECLS-B

**Fig. 4:**
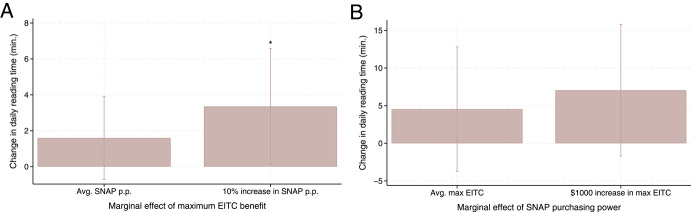
Marginal Effect of Each Program on Daily Reading Time by the Other Program’s Generosity Notes: The average SNAP purchasing power is 0.71; the average maximum federal and state EITC is $5830. Whether the marginal effects of EITC or SNAP statistically significantly change when the level of the other program’s generosity increases are indicated by the interaction term in Table 5. Stars above confidence intervals show the statistical significance of the marginal effect of each program. † p < 0.1, * p < 0.05, ** p < 0.01, *** p < 0.001. Data come from wave 3 and wave k in the ECLS-B (N = 1250)

An interesting result is found on parent’s time devoted to enriching activities with the child. While the main effects and the interaction effects on cognitive activity index (which comprises singing songs, storytelling, and reading with the child) are not statistically significant, I found that the interaction effect on daily reading time with the child is positive (2.51 min) and statistically significant. Figure 4 shows the marginal effects of each program, generated from this regression model. Panel A illustrates that, for children exposed to a 10% increase in SNAP purchasing power from its baseline mean, a $1000 increase in maximum EITC benefits results in a statistically significant increase in daily reading time by around 3.5 min (13% increase, relative to the sample mean of 25.4 min). On the contrary, for children exposed to the average SNAP purchasing power, a $1000 increase in maximum EITC benefits is associated with a smaller (by 1.6 min) and statistically nonsignificant increase in daily reading time. I found a similar trend in the marginal effects of SNAP purchasing power (though with less precise estimates), as shown in Panel B.

With respect to healthy eating, the main effect of SNAP purchasing power is large: a 0.1 increase in SNAP purchasing power is significantly associated with a 0.73 SD increase in health eating for children exposed to the average maximum EITC benefits. Though marginally significant, the interaction effect is also positive with a magnitude of 0.27 SD. This indicates that, with a $1,000 increase in maximum EITC benefits from the baseline mean, a 0.1 increase in SNAP purchasing power is associated with a 1.00 SD (rather than a 0.73 SD) improvement in healthy eating, suggesting there may be complementary changes in healthy eating. On the other hand, the main effect of maximum EITC benefits is small and not statistically significant.

## Discussion and Conclusion

This paper presents novel evidence that the generosity of EITC and SNAP purchasing power interact complementarily to shape cognitive development in the preschool to kindergarten-entry period. Using children’s differential exposure to SNAP purchasing power levels and maximum EITC benefit levels depending on region, year, and number of children in household, I found that the associations between maximum EITC benefits and early reading and early math skills are amplified for children exposed to greater SNAP purchasing power, and vice versa, with similar results found for the interactions between EITC purchasing power and SNAP purchasing power. Specifically, a $1000 increase in maximum EITC benefits led to 0.19–0.20 SD increases in early reading and math scores with a 10% increase in SNAP purchasing power from its baseline mean. To put these magnitudes into context, 0.19 SD-0.20 SD increases are comparable to closing 16–20% of the gap in math and reading scores at kindergarten-entry between children in the bottom and the top 10% of the income spectrum (Reardon et al., 2016). When compared to the effects of schooling, an increase of 0.19 SD in early math skills is comparable to the impact of attending state pre-kindergarten for eight months (Wong et al., 2008). I found larger magnitudes in the associations between SNAP purchasing power and those outcomes: they ranged between 0.35 SD-0.44 SD improvements, with a $1000 increase in maximum EITC benefits from the baseline mean.

These results suggest that one program becomes effective at reducing the negative effect of poverty on child development when coupled with more generous benefits from the other program. Given the close link of early cognitive outcomes with academic achievement and adult economic and health outcomes (Braak et al., 2022; Feinstein, & Duckworth, 2006; La-Scherban et al., 2014; Rabiner et al., 2016), this short-term effect may have longer term implications on children’s academic, health, and socioeconomic trajectories, potentially reducing economic inequality in child well-being. Overall, this study contributes to the emerging but still scarce literature on how multiple programs can interact to affect child and family wellbeing.

The test of mechanisms provided helpful, though preliminary, insights. I showed that there is a significant association between SNAP purchasing power and healthy eating – but not between maximum EITC benefits and healthy eating. Qualitative studies also suggest that EITC refunds are often spent on child-related expenses, including school supplies (Mendenhall et al., 2012; Romich & Weisner, 2000). Together, these findings point to the possibility that SNAP and EITC may affect distinct domains of well-being (e.g., nutrition and education), potentially leading to their positive interaction effects on early cognitive development. Findings also indicated that complementary changes in daily reading time may serve as another underlying mechanism. To better understand this time pathway, future research should explore consumption responses among families receiving both benefits, incorporating a broader set of variables such as transportation-related expenses (e.g., car repairs, vehicle purchases) and time-saving home appliances. Additionally, while the result on maternal depressive symptoms was statistically nonsignificant, I cannot definitively rule out the possibility of the family stress pathway as there may be other mediators not captured in the current study, such as maintaining an orderly household, close parental supervision, emotional support for the child, and the quality of parent–child interactions. In future research, it will be critical to more rigorously evaluate the specific mechanisms that underlie the positive interaction effects of EITC and SNAP, using a more comprehensive set of potential mediators and a formal mediation analysis.

Somewhat unexpectedly, I found that the main effect of each program at the average benefit level of the other program is statistically nonsignificant. A few possible explanations warrant further research. First, regarding the main effect of EITC, the amounts of EITC refunds or EITC-induced earnings received by families in the study sample may not have been large enough to generate measurable improvements in early cognitive outcomes. Previous studies show that a $1,000 increase in maximum EITC benefits translates into about $400 of actual EITC benefits (in 2021 dollars) (Maxfield, 2015). While their impacts on earnings are typically larger,^8^ because at least one parent was already working in the study sample, the labor-supply response would likely have been much smaller, meaning that increases in family resources would have come primarily from EITC refunds. Nevertheless, the 95% confidence intervals of the EITC’s main effect on reading or math still overlap, or come close to overlapping, with estimates from prior research—for example, Bastian and Michelmore (2018), who found that a $1,000 increase in maximum federal and state EITC benefits is associated with a 0.13 SD increase in test scores. The large standard error of the main effect of EITC may reflect limited variation in maximum EITC benefits.

The main effect of SNAP purchasing power is also statistically nonsignificant but has a larger magnitude than that of maximum EITC benefit. Its larger magnitude may reflect the different amount of change in family income, considering that a 0.1 increase in SNAP purchasing power (relative to the baseline mean) is comparable to $1344-$1739 (in 2021 dollars). However, the effect size of SNAP purchasing power should be interpreted with caution, because it is not consistent across alternative samples.

There are a few other limitations in this paper. First, due to data limitations, I could not fully capture families participating in EITC and SNAP. Without access to a family’s EITC receipt status, I focused on working families who receive SNAP, assuming a majority of them would have received EITC benefits. While EITC take up rates (around 80%) are higher than other programs (Tax Policy Center, 2019), measurement error may still be present in my sample. By using the self-reported SNAP receipt variable, my analysis may also be subject to endogenous underreporting problem (Bitler, 2020). However, both concerns are alleviated to some extent, because different types of study samples point towards a substantively similar result in most cases. Second, I cannot completely rule out the possibility of time-varying confounding factors, and to the extent that this is true, my estimates could have been biased to some degree. Third, while this study provides the first evidence on positive interaction effects of EITC and SNAP on early cognitive development, further considerations are needed to draw firm conclusions. In particular, EITC, SNAP, other safety net programs (e.g., CTC), as well as economic contexts have undergone changes since the study period, with more states adopting their own EITC program and the federal government expanding EITC in 2009. As such, future research should use more recent data to examine interaction effects of EITC and SNAP on cognitive outcomes, as well as other developmental domains, such as physical or mental health.

Fifth, although the EITC and SNAP benefit amount and eligibility are not directly affected by one another, it is still possible that they indirectly influence each other through labor supply changes over a longer period. Research shows that receipt of the EITC refunds serves as a work incentive in the longer-term (Bastian et al., 2022), although it may serve as a temporary work disincentive immediately after the receipt of refunds (because it increases resources), especially for married women (LaLumia, 2013; Yang, 2017). While - as noted above - the labor supply increase in response to EITC would likely have been small in our sample of already working families, any increases in labor supply will increase income and may reduce the amount of SNAP benefits a family is eligible for or lead families to not participate in SNAP. For instance, Mikelson and Lerman (2004) found some evidence of a negative impact of EITC benefits on SNAP participation, but this effect was mixed across different models. Similarly, if SNAP benefits reduce labor supply – but importantly, there are mixed findings on the labor supply effect of SNAP – this will reduce earnings and can potentially reduce the amount of EITC refunds received in the following calendar year (when they file taxes for the current tax year), depending on the family’s earnings level. If families decide to leave the labor market, they will become ineligible for EITC.

Such dynamics between EITC and SNAP are less of a concern at the extensive margin (i.e., the SNAP or EITC *receipt status* being affected by one another), because the study sample is restricted to families who reported that they received SNAP in both study waves. Also, the majority of mothers or resident fathers who worked in wave 2 (which represents, although not perfectly, their work status in the tax year relevant to the EITC received in wave 3) continued to work in later waves (76.5% and 80.4% of them continued to work in wave 3 and wave k, respectively).

On the other hand, such dynamics between EITC and SNAP may show up at the intensive margin (i.e., their *benefit amount* being affected by one another). As the reduction in benefit amount is not accounted for by the maximum EITC benefit or SNAP purchasing power, the actual benefits received by families in wave k may have been lower than the expected amount based on those measures. To the extent that the amount of reduction is substantial, the study’s estimates of positive interaction effects could have been underestimated.

The study has timely policy implications. In particular, findings from this study should be carefully taken into account when policymakers evaluate the cost-effectiveness of any proposed changes to the SNAP and EITC budgets. First, to benefit from complementary effects of EITC and SNAP in the first place, it is important that all eligible families and children participate in both programs. Although the take up rates are higher in the EITC and SNAP compared to other safety net benefits (76% to 81% of SNAP take-up rates and 81% of federal EITC take-up rates during the study period: see Tax Policy Center, 2019), still, not all eligible families are receiving them partly due to administrative burdens (Moynihan et al., 2015). Therefore, to maximize the effectiveness of these programs, it would be critical to simplify the SNAP application and recertification process and the tax filing process. For instance, one approach could include creating a “non-filer online portal” for those who are not required to file tax returns. Policymakers could also consider increasing government funding to community organizations involved in public outreach, such as SNAP Outreach program and Volunteer Income Tax Assistance program. These agencies could help families understand the application process, fill out necessary forms, and gather required documentation.

Second, there are still many states that have not yet implemented refundable state EITC policies. This study suggests that adopting and expanding state EITCs – which would increase the amount of EITC benefits that an eligible family receives – could enhance the effectiveness of EITC in promoting children’s early cognitive development, particularly when they are combined with greater SNAP benefits. Third, using the SNAP purchasing power approach, I demonstrated the existence of disparities in SNAP purchasing power across regions. This would prevent low-income families and their children from equally benefiting from SNAP, and thereby weaken the effectiveness of SNAP in improving children’s early cognitive development. Hence, addressing such disparities, such as by raising SNAP benefits to reflect local prices, would be important, particularly in light of the current period of high inflation. Providing state- or local- level SNAP benefits is another way to reduce regional disparities in SNAP purchasing power and enhance the effectiveness of SNAP, as unlike other large anti-poverty programs, such as EITC, states do not offer their own SNAP benefit program.

These policy implications may extend to other countries. Confirming this would require further evidence, however, as there is relatively little research globally – similar to the U.S. – on patterns of multiple program participation (see Wu et al., 2025 as an example) and the potential interaction effects of multiple safety net programs on child and family well-being. Given that many countries offer multiple programs, this study highlights the need for more comprehensive analyses of multiple social safety net benefits in diverse countries.

## Supplementary Information

Below is the link to the electronic supplementary material.

Supplementary file1 (DOCX 789 kb)

## Footnotes

1 This is based on EITC eligibility, assuming 100% participation.

2 States have the option to increase the gross income limit up to 200% of the FPL by adopting the broad-based categorical eligibility. During the study period (2004–2007), up to 13 states have implemented this policy.

3 In this measure, I do not differentiate between refundable and non-refundable state EITCs. However, results are nearly unchanged when I only consider refundable state EITCs.

4 Since moving could be endogenous, I use state of residence in wave 2, measured prior to the study period. In the main sample, I do not restrict the sample based on whether families moved, but I find qualitatively similar results (available upon request) when I only consider families who did not move to different states from wave 2 to wave k.

5 The increase in the standardized value of early math outcomes does not seem to affect the study results. Findings are substantively similar in alternative samples (e.g., SNAP-receiving households overall) where early math outcomes do not statistically significantly increase across waves (available upon request).

6 When SNAP purchasing power increases by 10% from the mean (0.71), it results in 0.78, which is the highest SNAP purchasing power level among market groups during the study period. 0.71 is calculated by dividing the average maximum SNAP benefit per person – i.e., $122 – by the average monthly TFP cost per person – i.e., $174. Therefore, to increase the average SNAP purchasing power by 10% from the mean, it would require an increase in maximum SNAP benefits by approximately $12 or a decrease in TFP cost by $17 per person-month. If we translate them into annual dollar amounts for an average household size (4.9) in the study sample, a 10% increase indicates an additional $706-$1000 per year (approximately $949-$1344 in 2021 dollars).

7 WIC is another program that SNAP participants may have also been enrolled in. However, as children are eligible for WIC up to age 5, close to 90% of those surveyed in wave 5 (whose average age is 74 months) were not asked about their WIC receipt.

8 Bastian and Michelmore (2018), using both federal and state policy EITC policy changes, found that a $1000 increase in maximum EITC benefits led to a $3250 increase in family earnings (in 2021 dollars), which was mostly driven by increases in maternal labor market participation rate (at the extensive margin).
