# Supplementary material for: Do Larger Earned Income Tax Credit and Supplemental Nutrition Assistance Program Benefits Create Complementary Effects on Child Development?
Source: Popul Res Policy Rev. 2026 Jan 22;45(1):5. doi: 10.1007/s11113-025-09985-9 (PMC12827319; doi:10.1007/s11113-025-09985-9)
Supplement: Supplementary file 1 — Supplementary file1 (DOCX 789 kb) [file 11113_2025_9985_MOESM1_ESM.docx]

**ONLINE SUPPLEMENT**

**Do Larger Earned Income Tax Credit and Purchasing Power of Supplemental Nutrition Assistance Program Benefits Create Complementary Effects on Child Development?**

| **Table S1.** States with Changes in their EITC Generosity or with Different Levels of EITC Generosity by the Number of Children | | | | |
| --- | --- | --- | --- | --- |
| *Panel A. States that increased their state EITC generosity* | | | |  |
| State | Tax year | State credit rate | Refundability | In the sample |
| DC | 2003 | 0.25 | 1 | N |
| DC | 2004 | 0.25 | 1 | N |
| DC | 2005 | 0.35 | 1 | N |
| DC | 2006 | 0.35 | 1 | N |
|  |  |  |  |  |
| DE | 2003 | 0 | 0 | N |
| DE | 2004 | 0 | 0 | N |
| DE | 2005 | 0 | 0 | N |
| DE | 2006 | 0.2 | 0 | N |
|  |  |  |  |  |
| MD | 2003 | 0.18 | 1 | Y |
| MD | 2004 | 0.2 | 1 | Y |
| MD | 2005 | 0.2 | 1 | Y |
| MD | 2006 | 0.2 | 1 | Y |
|  |  |  |  |  |
| NE | 2003 | 0 | 0 | Y |
| NE | 2004 | 0 | 0 | Y |
| NE | 2005 | 0 | 0 | Y |
| NE | 2006 | 0.08 | 1 | Y |
|  |  |  |  |  |
| OR | 2003 | 0.05 | 0 | N |
| OR | 2004 | 0.05 | 0 | N |
| OR | 2005 | 0.05 | 0 | N |
| OR | 2006 | 0.05 | 1 | N |
|  |  |  |  |  |
| RI | 2003 | 0.05 | 1 | Y |
| RI | 2004 | 0.05 | 1 | Y |
| RI | 2005 | 0.1 | 1 | Y |
| RI | 2006 | 0.15 | 1 | Y |
|  |  |  |  |  |
| VA | 2003 | 0 | 0 | N |
| VA | 2004 | 0 | 0 | N |
| VA | 2005 | 0 | 0 | N |
| VA | 2006 | 0.2 | 0 | N |
|  |  |  |  |  |
| *Panel B. States with different state credit rates by the number of children during tax years 2003-2006.* | | | | |
| State | Number of children | State credit rate | Refundability | In the sample |
| MN | 1 | 0.3 | 1 | Y |
| MN | 2 | 0.35 | 1 | Y |
| MN | 3+ | 0.35 | 1 | Y |
|  |  |  |  |  |
| WI | 1 | 0.04 | 1 | Y |
| WI | 2 | 0.14 | 1 | Y |
| WI | 3+ | 0.43 | 1 | Y |
| Notes: National Bureau of Economic Research [NBER], 2019) and Komro et al. (2020). "N" stands for No, "Y" stands for Yes. | | | | |

| **Table S2.** Relationship Between SNAP Purchasing Power and SNAP Participation among Likely SNAP-Eligible Households | | | | |
| --- | --- | --- | --- | --- |
|  | Unmarried, high school degree or below | | Low-income households (<200% FPL) | |
|  | Wave 3 to Wave K | Wave 1 to Wave K | Wave 3 to Wave K | Wave 1 to Wave K |
| SNAP purchasing power | 0.03 | -0.07 | 0.00 | -0.03 |
|  | (0.08) | (0.06) | (0.05) | (0.03) |
|  |  |  |  |  |
| N | 1250 | 2800 | 5100 | 11150 |
| Notes: All estimates are weighted, except for sample sizes. Only the key coefficients are displayed, but all covariates are controlled, including child and year fixed effects, state-level economic and policy characteristics, local prices of other goods, and child or family level characteristics. The first sample (in columns 1-2) comprises mothers who were unmarried and had high school degree or below across wave 2 to wave k. The second sample (in columns 3-4) includes households with income below 200% of the federal poverty line (FPL) based on wave 2 information. † p<0.1, * p<0.05, ** p<0.01, *** p<0.001. Data come from the ECLS-B. | | | | |

| **Table S3.** Relationship Between Thrifty Food Plan Price and Other Prices | | |
| --- | --- | --- |
|  | (1) | (2) |
|  | TFP price | TFP price |
| Regional CPI for apparel costs | -0.10*** | 0.01*** |
|  | (0.01) | (0.00) |
| Regional CPI for education costs | 0.75*** | -0.02** |
|  | (0.03) | (0.01) |
| Regional CPI for recreation costs | -0.34*** | 0.06*** |
|  | (0.03) | (0.01) |
| Regional CPI for transportation costs | 0.11*** | -0.01*** |
|  | (0.01) | (0.00) |
| Fair market rent for 2-bedroom | 0.04*** | 0.00 |
|  | (0.00) | (0.00) |
|  |  |  |
| Market group FE | N | Y |
| Year FE | N | Y |
| N | 12394 | 12394 |
| R-squared | 0.48 | 0.98 |
| Notes: This table is generated from regression models of TFP price (which is used to construct the SNAP purchasing power measure) on other price measures. † p<0.1, * p<0.05, ** p<0.01, *** p<0.001. Data come from the county-year level data on SNAP purchasing power from 2004 to 2007. | | |

| **Table S4.** Comparing Households with Changes in the Number of Children Between One Child and Two or More Children Versus Other Households | | | |
| --- | --- | --- | --- |
|  | Mean(SD) / Percent | |  |
|  | Other households (N=1200) | Households with changes in the number of children between one child and two or more children (N=100) | Sig. |
| Married (%) (Ref.: Not married) | 42.35 | 34.56 |  |
| Urbanicity (%) (Ref.: Rural) |  |  |  |
| Urban | 60.69 | 73.52 |  |
| Urban-cluster | 15.12 | 12.64 |  |
| Parent's highest education attainment (%) (Ref.: No high school degree) |  |  |  |
| High school degree | 41.23 | 55.21 |  |
| Some college | 34.12 | 21.91 |  |
| BA or higher | 4.42 | 4.55 |  |
| Received Medicaid (%) (Ref.: Did not receive) | 87.88 | 93.4 |  |
| Child race/ethnicity (%) (Ref.: White) |  |  |  |
| Black | 32.89 | 31.09 |  |
| Hispanic | 21.95 | 17.84 |  |
| Others | 6.28 | 13.41 |  |
| Child is male (%) (Ref.: female) | 48.64 | 47.93 |  |
| Poverty status (%) (Ref.: <50% of FPL) |  |  | † |
| 50%-130% of FPL | 57.61 | 51.32 |  |
| >130% of FPL | 15.08 | 27.92 |  |
| Mother is employed (%) (Ref.: Not employed) | 50.50 | 50.25 |  |
| Child age at the interview (in months) | 60.13 | 60.38 |  |
|  | (8.94) | (10.05) |  |
| Mother's age (in years) | 29.86 | 26.90 |  |
|  | (6.28) | (5.68) |  |
| Notes: All estimates are weighted, except for sample sizes. 'Sig.' column shows whether a given variable is statistically significantly different between the two groups. Chi-square tests are used to test the differences in categorical variables and t-tests are used to test the differences in continuous variables. † p<0.1, * p<0.05, ** p<0.01, *** p<0.001. Data come from wave 3 and wave k in the ECLS-B (N=1300). | | | |

| **Table S5.** Interaction Effects Between Maximum EITC and SNAP Purchasing Power, Removing Families with Changes in Births between One Child and Two or More Children | | |
| --- | --- | --- |
|  | Reading | Math |
| Maximum EITC (centered) | -0.06 | 0.05 |
|  | (0.21) | (0.27) |
| SNAP purchasing power (centered) | 0.20 | 0.35* |
|  | (0.18) | (0.17) |
| Maximum EITC $\times$SNAP purchasing power | 0.30* | 0.32* |
|  | (0.12) | (0.13) |
| Notes: All estimates are weighted, except for sample sizes. Only the key coefficients are displayed, but all covariates are controlled, including child and year fixed effects, state-level economic and policy characteristics, local prices of other goods, and child or family level characteristics. † p<0.1, * p<0.05, ** p<0.01, *** p<0.001. Data come from wave 3 and wave k in the ECLS-B (N=1200). | | |

| **Table S6.** Main Effects of Maximum EITC and SNAP Purchasing Power without their Interaction Term | | |
| --- | --- | --- |
|  | Reading | Math |
| Maximum EITC | 0.04 | -0.01 |
|  | (0.06) | (0.05) |
| SNAP purchasing power | 0.16 | 0.21 |
|  | (0.19) | (0.18) |
| Notes: All estimates are weighted, except for sample sizes. Only the key coefficients are displayed, but all covariates are controlled, including child and year fixed effects, state-level economic and policy characteristics, local prices of other goods, and child or family level characteristics. † p<0.1, * p<0.05, ** p<0.01, *** p<0.001. Data come from wave 3 and wave k in the ECLS-B (N=1300). | | |

| **Table S7.** Interaction Effects Between EITC Purchasing Power and SNAP Purchasing Power | | | | |
| --- | --- | --- | --- | --- |
|  | Working families | No deep poverty | Unmarried, High school or below | Overall SNAP households |
| Panel A. Early reading skills |  |  |  |  |
| EITC purchasing power (centered) | 0.09 | 0.11* | -0.06 | 0.06 |
|  | (0.08) | (0.05) | (0.07) | (0.05) |
| SNAP purchasing power (centered) | 0.21 | 0.12 | 0.13 | -0.00 |
|  | (0.19) | (0.19) | (0.19) | (0.18) |
| EITC purchasing power $\times$ SNAP purchasing power | 0.25** | 0.38*** | 0.12 | 0.19** |
|  | (0.08) | (0.07) | (0.07) | (0.05) |
| Panel B. Early math skills |  |  |  |  |
| EITC purchasing power (centered) | 0.03 | 0.03 | 0.03 | 0.01 |
|  | (0.07) | (0.06) | (0.09) | (0.05) |
| SNAP purchasing power (centered) | 0.28 | 0.15 | 0.15 | 0.13 |
|  | (0.19) | (0.15) | (0.16) | (0.15) |
| EITC purchasing power $\times$ SNAP purchasing power | 0.26* | 0.34*** | 0.23** | 0.26** |
|  | (0.11) | (0.09) | (0.08) | (0.07) |
| N | 1300 | 1350 | 1250 | 2050 |
| Notes: EITC purchasing power measure is calculated as the ratio of the maximum federal and state EITC benefits to the market group level TFP price. A unit increase is 0.1 in both the EITC and SNAP purchasing power measures. Each panel is a separate regression, and within each panel, four regressions are run by different samples. In all panels, only the key coefficients are displayed, but all covariates are controlled, including child and year fixed effects, state-level economic and policy characteristics, local prices of other goods, and child or family level characteristics. All estimates are weighted, except for sample sizes. † p<0.1, * p<0.05, ** p<0.01, *** p<0.001. Data come from wave 3 and wave k in the ECLS-B. | | | | |

| **Table S8.** Effects of SNAP Purchasing Power on Household Income to Needs Ratio and Income | | |
| --- | --- | --- |
|  | Income to needs ratio | Household income (in $2021) |
| SNAP purchasing power | -0.18 | -2888.33 |
|  | (0.11) | (3148.44) |
| Notes: All estimates are weighted, except for sample sizes. Only the key coefficient is displayed, but all covariates are controlled, including child and year fixed effects, state-level economic and policy characteristics, local prices of other goods, and child or family level characteristics (however, results do not change without the state-level controls, other prices, and individual characteristics; available upon request). † p<0.1, * p<0.05, ** p<0.01, *** p<0.001. Data come from wave 3 and wave k in the ECLS-B (N=1300). | | |

| **Table S9.** Controlling for Participation in Other Cash-Based Programs | | |
| --- | --- | --- |
|  | Reading | Math |
| Maximum EITC (centered) | 0.08 | 0.04 |
|  | (0.06) | (0.06) |
| SNAP purchasing power (centered) | 0.19 | 0.24 |
|  | (0.18) | (0.18) |
| Maximum EITC $\times$SNAP purchasing power | 0.18** | 0.22* |
|  | (0.05) | (0.08) |
| Received TANF | 0.10 | 0.10 |
|  | (0.08) | (0.08) |
| Received SSDI or SSI | -0.25 | -0.25 |
|  | (0.22) | (0.17) |
| Notes: All estimates are weighted, except for sample sizes. In both models, all covariates are controlled, including child and year fixed effects, state-level economic and policy characteristics, local prices of other goods, and child or family level characteristics. † p<0.1, * p<0.05, ** p<0.01, *** p<0.001. Data come from wave 3 and wave k in the ECLS-B (N=1300). | | |

| **Table S10.** Demographic Characteristics by Different Assessment Timing Groups | | | | | | | |
| --- | --- | --- | --- | --- | --- | --- | --- |
|  | 2005-2006 | 2005-2007 | 2006-2007 | 2006-2006 | 2005-2008 | 2006-2008 | Sig. |
| Household size (%)  (Ref.: Two) |  |  |  |  |  |  | * |
| Three | 10.88 | 16.12 | 18.81 | 28.14 | 33.34 | 37.83 |  |
| Four | 29.58 | 28.53 | 9.08 | 12.68 | 9.26 | 16.95 |  |
| Five | 27.05 | 22.86 | 22.04 | 29.12 | 17.37 | 21.39 |  |
| Six or more | 26.52 | 23.75 | 50.07 | 20.51 | 40.02 | 23.83 |  |
| Two or more children (%) (Ref.: Family with one child) | 83.95 | 83.08 | 96.81 | 87.44 | 88.3 | 83.29 |  |
| Married (%)  (Ref.: Not married) | 41.7 | 42.05 | 59.39 | 34.41 | 29.77 | 15.52 |  |
| Urbanicity (%) (Ref.: Rural) |  |  |  |  |  |  |  |
| Urban/Urban-cluster | 78.71 | 71.63 | 89.08 | 69.35 | 85.69 | 72.25 |  |
| Parent's highest education attainment (%)  (Ref.: No high school degree) |  |  |  |  |  |  | * |
| High school degree | 48.33 | 36.16 | 37.07 | 39.99 | 19.89 | 53.26 |  |
| Some college/BA or higher | 35.44 | 32.29 | 41.81 | 49.92 | 68.1 | 34.98 |  |
| Child race/ethnicity (%)  (Ref.: White) |  |  |  |  |  |  |  |
| Black | 33.32 | 26.5 | 28.02 | 44.52 | 49.36 | 44.91 |  |
| Hispanic/Others | 30.61 | 25.13 | 36.5 | 21.85 | 29.35 | 15.1 |  |
| Poverty status (%)  (Ref.: <50% of FPL) |  |  |  |  |  |  |  |
| 50%-130% of FPL | 55.92 | 58 | 63.41 | 50.93 | 59.96 | 59.46 |  |
| >130% of FPL | 16.49 | 17.5 | 7.61 | 22.3 | 19.23 | 12.66 |  |
| Received Medicaid (%)  (Ref.: Did not receive) | 86.42 | 87.9 | 93.08 | 93.17 | 100 | 100 |  |
| Sample size of each group | 750 | 300 | 100 | 100 | 50 | NA |  |
| Notes: Sample size for the ‘2006-2008’ group is NA because the reporting standards were not met. For a few variables (urbanicity, education attainment, and child race/ethnicity), to meet the reporting standards, a category with a small number of respondents was combined with other category. Those categories include urban-cluster (combined with urban), BA or higher (combined with some college), and other race/ethnicity (combined with Hispanic). All estimates are weighted, except for sample size. Chi-square tests are used to test the differences in categorical variables and t-tests are used to test the differences in continuous variables. The p-value from these tests is shown in the ‘Sig.’ column. † p<0.1, * p<0.05, ** p<0.01, *** p<0.001. Data come from wave 3 and wave k in the ECLS-B. | | | | | | | |

**Table S11.** Construction of Mediators Considered in the Test of Mechanisms

| Variables | How they are constructed | Mean (SD) /  [Range] /  Percentages (%) |
| --- | --- | --- |
| Mother’s depressive symptoms | To construct the indicator for whether a mother has depressive symptoms, this paper used a self-reported depression scale in the ECLS-B data. The ECLS-B used a 12-item version of the Center for Epidemiological Studies’ Depression Scale (Radloff, 1977), which assesses depressive feelings and behavior during the past week. Each item is coded on a 4-point scale between 0 and 3, and total scores of all items are categorized into no (0-4), mild (5-9), moderate (10-14), or severe depressive symptoms (15+) (Paulson, Keefe, & Leiferman, 2009; Silverstein et al., 2006). Mothers having moderate or severe depressive symptoms are coded as 1 for this indicator. | Moderately or severely depressed (30.65%), Not, or mildly depressed (69.35%) |
| Mother’s severe depressive symptoms | I use the same scale above to construct the indicator for having severe depressive symptoms. | Severely depressed (14.19%), Not, mildly, or moderately depressed (85.81%) |
| Number of books | This is a continuous variable that measures the number of books at home. In the analysis, it is standardized to have a mean of zero and a standard deviation of 1. | -0.30 (0.59) [-0.79, 9.33] |
| Cognitive activity index | This is calculated by averaging the values of the following three items, which are coded on a 4-point scale (1 “not at all”, 2 “once or twice” 3 “3 to 6 times” 4 “every day”): how often mothers or any other family members read books to the child, sing songs with the child, and tell stories to the child in a typical week. | 2.87 (0.69) [1.00, 4.00] |
| Reading time | This is a continuous variable that measures the minutes per day that mothers or other family members read to the child. | 25.44 (20.56) [0.00, 300.00] |
| Healthy eating index | This is calculated by averaging the values of four items that indicate the frequency that the child drank milk, drank 100% fruit juice, ate vegetables, and ate fruit during the past 7 days. Each item is assessed on a 7-point scale, and I convert this into a continuous scale that measures an approximate frequency of consuming each item per week: “none” (~assigned with 0); “one to three times during the past 7 days” (~assigned with 2); “four to six times during the past 7 days” (~assigned with 5); “once a day” (~assigned with 7); “twice a day” (~assigned with 14); “three times a day” (~assigned with 21); “four or more times a day” (~assigned with 28). Finally, the averaged value is standardized to have a mean of zero and a standard deviation of 1. | 0.07 (1.08) [-2.19, 3.88] |

Notes: All estimates are weighted. Data come from wave 3 and wave k in the ECLS-B.

| **Table S12.** Full Regression Results of the Interaction Effects between the Maximum EITC and the SNAP purchasing power. | | | | | | | | | | |
| --- | --- | --- | --- | --- | --- | --- | --- | --- | --- | --- |
|  | Reading | | | | | Math | | | | |
|  | Main | No deep poverty | Overall SNAP | Unmarried, high school or below | Married, College degree | Main | No deep poverty | Overall SNAP | Unmarried, high school or below | Married, College degree |
| Maximum EITC (centered) | 0.07 | 0.09† | 0.05 | -0.05 | -0.05 | 0.04 | 0.03 | 0.02 | 0.04 | -0.04 |
|  | (0.06) | (0.05) | (0.04) | (0.06) | (0.04) | (0.06) | (0.05) | (0.04) | (0.07) | (0.04) |
| SNAP purchasing power (centered) | 0.17 | 0.02 | -0.05 | 0.04 | -0.15 | 0.22 | 0.02 | 0.04 | 0.03 | -0.07 |
|  | (0.18) | (0.18) | (0.19) | (0.19) | (0.15) | (0.18) | (0.13) | (0.15) | (0.16) | (0.11) |
| Maximum EITC X SNAP purchasing power | 0.18** | 0.26*** | 0.12** | 0.05 | -0.01 | 0.21* | 0.26*** | 0.21*** | 0.16** | 0.02 |
|  | (0.05) | (0.06) | (0.04) | (0.05) | (0.05) | (0.08) | (0.06) | (0.06) | (0.05) | (0.04) |
| Wave K | -0.90*** | -0.94*** | -0.90*** | -1.02*** | -1.24*** | -1.21*** | -1.14*** | -1.18*** | -1.29*** | -1.29*** |
|  | (0.17) | (0.18) | (0.16) | (0.12) | (0.08) | (0.14) | (0.12) | (0.12) | (0.14) | (0.07) |
| Unemployment rate | -0.02 | 0.13† | -0.01 | 0.04 | -0.04 | -0.10 | -0.02 | -0.05 | -0.08 | -0.01 |
|  | (0.06) | (0.07) | (0.06) | (0.06) | (0.07) | (0.08) | (0.08) | (0.07) | (0.07) | (0.07) |
| Percent of poverty | -0.04 | -0.07 | -0.05 | -0.16* | -0.01 | -0.03 | 0.01 | -0.04 | -0.09 | -0.00 |
|  | (0.06) | (0.06) | (0.05) | (0.06) | (0.04) | (0.08) | (0.08) | (0.07) | (0.09) | (0.06) |
| Per-capita income ($2021 in thousands) | 0.05 | 0.03 | -0.00 | 0.00 | 0.04 | -0.00 | -0.02 | -0.02 | -0.05 | 0.02 |
|  | (0.04) | (0.03) | (0.04) | (0.05) | (0.03) | (0.03) | (0.04) | (0.03) | (0.05) | (0.03) |
| Maximum TANF benefits for a family of four ($2021 in hundreds) | 0.20 | 0.23 | 0.12 | 0.01 | 0.19 | 0.04 | 0.01 | -0.01 | -0.12 | 0.03 |
|  | (0.17) | (0.18) | (0.16) | (0.13) | (0.12) | (0.21) | (0.21) | (0.18) | (0.12) | (0.07) |
| Medicaid income eligibility limit as a percent of FPL | -1.79*** | -1.80** | -0.75** | -0.33 | -0.92*** | -0.30 | -0.32 | 0.45* | 1.53*** | 0.14 |
|  | (0.46) | (0.51) | (0.22) | (0.22) | (0.13) | (0.89) | (1.19) | (0.21) | (0.16) | (0.12) |
| Minimum wage ($2021) | -0.18** | -0.16** | -0.13* | -0.09† | -0.09** | -0.03 | -0.05 | -0.05 | -0.09† | -0.03 |
|  | (0.06) | (0.05) | (0.05) | (0.05) | (0.03) | (0.06) | (0.05) | (0.05) | (0.05) | (0.03) |
| Regional CPI for apparel costs | 0.03* | 0.02 | 0.01 | 0.01 | 0.00 | 0.03** | 0.02 | 0.01 | 0.00 | -0.00 |
|  | (0.01) | (0.01) | (0.01) | (0.01) | (0.01) | (0.01) | (0.01) | (0.01) | (0.01) | (0.01) |
| Regional CPI for education costs | 0.05* | 0.07** | 0.04 | 0.01 | 0.02 | 0.04* | 0.02 | 0.03† | 0.02 | 0.04* |
|  | (0.02) | (0.02) | (0.03) | (0.03) | (0.01) | (0.02) | (0.01) | (0.02) | (0.01) | (0.01) |
| Regional CPI for recreation costs | 0.04 | 0.04 | 0.02 | 0.04 | -0.01 | 0.02 | 0.02 | 0.01 | 0.01 | -0.03 |
|  | (0.03) | (0.03) | (0.03) | (0.02) | (0.02) | (0.02) | (0.02) | (0.02) | (0.02) | (0.02) |
| Regional CPI for transportation costs | -0.03* | -0.01 | -0.01 | -0.00 | 0.02† | -0.00 | -0.01 | -0.01 | 0.01 | 0.01 |
|  | (0.01) | (0.01) | (0.01) | (0.01) | (0.01) | (0.01) | (0.01) | (0.01) | (0.01) | (0.01) |
| Fair market rent for 2-bedroom ($2021) | -0.00 | -0.00 | -0.00 | 0.00 | 0.00 | -0.00 | -0.00 | -0.00 | 0.00 | 0.00 |
|  | (0.00) | (0.00) | (0.00) | (0.00) | (0.00) | (0.00) | (0.00) | (0.00) | (0.00) | (0.00) |
| Child age at the interview | 0.07 | 0.10** | 0.05 | 0.00 | 0.09*** | 0.07 | 0.10† | 0.09† | 0.04 | 0.12*** |
|  | (0.06) | (0.03) | (0.03) | (0.06) | (0.02) | (0.07) | (0.05) | (0.04) | (0.07) | (0.03) |
| Squared child age at the interview | -0.00 | -0.00 | 0.00 | 0.00 | -0.00† | 0.00 | -0.00 | -0.00 | 0.00 | -0.00* |
|  | (0.00) | (0.00) | (0.00) | (0.00) | (0.00) | (0.00) | (0.00) | (0.00) | (0.00) | (0.00) |
| Mother's age | -0.04 | -0.03 | -0.03 | -0.04 | 0.40* | -0.01 | -0.00 | -0.04† | -0.05 | 0.34* |
|  | (0.03) | (0.03) | (0.02) | (0.04) | (0.17) | (0.04) | (0.02) | (0.02) | (0.03) | (0.16) |
| Squared mother's age | 0.00 | 0.00 | 0.00† | 0.00 | -0.00* | 0.00 | -0.00 | 0.00† | 0.00 | -0.00 |
|  | (0.00) | (0.00) | (0.00) | (0.00) | (0.00) | (0.00) | (0.00) | (0.00) | (0.00) | (0.00) |
| High school degree | 0.20* | 0.15 | 0.11 | -0.02 |  | 0.05 | 0.00 | 0.07 | -0.11* |  |
|  | (0.09) | (0.11) | (0.07) | (0.13) |  | (0.09) | (0.12) | (0.07) | (0.04) |  |
| Some College | 0.18 | 0.11 | 0.09 |  |  | -0.05 | -0.11 | -0.01 |  |  |
|  | (0.14) | (0.16) | (0.12) |  |  | (0.10) | (0.16) | (0.10) |  |  |
| BA or higher | 0.14 | 0.08 | -0.04 |  |  | -0.07 | -0.24 | -0.01 |  |  |
|  | (0.21) | (0.20) | (0.14) |  |  | (0.22) | (0.23) | (0.14) |  |  |
| Married | 0.00 | 0.14 | -0.01 |  |  | 0.19 | 0.03 | 0.01 |  |  |
|  | (0.15) | (0.12) | (0.11) |  |  | (0.12) | (0.13) | (0.10) |  |  |
| Urban | 0.41† | 0.53** | 0.40* | -0.58* | -0.00 | 0.41† | 0.59*** | 0.41** | -0.12 | 0.17 |
|  | (0.22) | (0.16) | (0.18) | (0.22) | (0.19) | (0.21) | (0.15) | (0.15) | (0.38) | (0.24) |
| Urban-cluster | 0.47 | 0.42 | 0.34 | -0.85* | 0.09 | 0.37 | 0.50† | 0.42† | -0.34 | -0.01 |
|  | (0.31) | (0.29) | (0.26) | (0.36) | (0.25) | (0.26) | (0.25) | (0.23) | (0.58) | (0.23) |
| Family of three | 0.12 | 0.21 | 0.13 | -0.10 | 0.80*** | -0.21 | -0.34† | -0.21 | -0.15 | 0.38 |
|  | (0.10) | (0.15) | (0.11) | (0.12) | (0.19) | (0.20) | (0.17) | (0.20) | (0.18) | (0.34) |
| Family of four | 0.16 | 0.24† | 0.20* | -0.08 | 0.77** | -0.09 | -0.16 | -0.08 | -0.20 | 0.35 |
|  | (0.11) | (0.13) | (0.09) | (0.15) | (0.23) | (0.22) | (0.21) | (0.23) | (0.17) | (0.43) |
| Family of five | 0.10 | 0.19 | 0.13 | -0.10 | 0.69* | -0.18 | -0.14 | -0.12 | -0.15 | 0.14 |
|  | (0.13) | (0.15) | (0.12) | (0.15) | (0.32) | (0.20) | (0.23) | (0.23) | (0.20) | (0.46) |
| Family of six or more | 0.28 | 0.16 | 0.16 | -0.19 | 0.84* | -0.08 | -0.07 | -0.02 | -0.17 | 0.15 |
|  | (0.18) | (0.15) | (0.14) | (0.15) | (0.38) | (0.19) | (0.21) | (0.24) | (0.19) | (0.44) |
| Received Medicaid | 0.01 | 0.10 | 0.08 | 0.08 | 0.07 | 0.14 | 0.11 | 0.13 | 0.14 | -0.23** |
|  | (0.12) | (0.11) | (0.09) | (0.11) | (0.13) | (0.10) | (0.13) | (0.09) | (0.09) | (0.08) |
| N | 1300 | 1350 | 2050 | 1250 | 3850 | 1300 | 1350 | 2050 | 1250 | 3850 |
| Notes: All estimates are weighted, except for sample sizes. The ‘Main’ column comprises SNAP households in which at least one parent worked in wave 2. † p<0.1, * p<0.05, ** p<0.01, *** p<0.001. Data come from wave 3 and wave k in the ECLS-B (N=1300). | | | | | | | | | | |

| **Table S13.** Full Regression Results from the Test of Mechanisms | | | | | | |
| --- | --- | --- | --- | --- | --- | --- |
|  | Severe depressive symptoms | Depressive symptoms | Books (std.) | Cognitive activity | Read time | Healthy eating (std.) |
| Maximum EITC (centered) | 0.02 | -0.02 | -0.04 | -0.04 | 1.60 | 0.05 |
|  | (0.03) | (0.03) | (0.03) | (0.05) | (1.12) | (0.08) |
| SNAP purchasing power (centered) | -0.06 | 0.05 | 0.24† | -0.18 | 4.55 | 0.73* |
|  | (0.09) | (0.11) | (0.14) | (0.15) | (4.22) | (0.32) |
| Maximum EITC X SNAP purchasing power | 0.00 | 0.03 | 0.02 | -0.10 | 2.51* | 0.27† |
|  | (0.04) | (0.04) | (0.06) | (0.07) | (1.22) | (0.15) |
| Wave K | -0.04 | 0.05 | -0.05 | -0.10 | 1.94 | -0.21 |
|  | (0.09) | (0.08) | (0.07) | (0.09) | (6.62) | (0.17) |
| Unemployment rate | -0.01 | -0.02 | 0.03 | 0.11* | 0.36 | 0.02 |
|  | (0.03) | (0.03) | (0.04) | (0.05) | (2.01) | (0.11) |
| Percent of poverty | 0.02 | 0.00 | 0.03 | -0.03 | 1.51 | -0.20 |
|  | (0.04) | (0.04) | (0.05) | (0.05) | (1.20) | (0.15) |
| Per-capita income ($2021 in thousands) | 0.02 | -0.01 | 0.02 | 0.00 | 0.15 | 0.03 |
|  | (0.02) | (0.02) | (0.03) | (0.03) | (0.56) | (0.06) |
| Maximum TANF benefits for a family of four ($2021 in hundreds) | 0.15 | 0.12 | -0.35 | -0.31 | -17.55 | -0.60 |
|  | (0.12) | (0.15) | (0.24) | (0.29) | (10.98) | (0.43) |
| Medicaid income eligibility limit as a percent of FPL | -0.39 | -0.73 | 0.03 | 0.15 | 22.31 | -0.56 |
|  | (0.25) | (0.49) | (0.72) | (0.64) | (15.37) | (1.12) |
| Minimum wage ($2021) | 0.00 | -0.04 | -0.05 | -0.08 | 0.88 | -0.04 |
|  | (0.03) | (0.05) | (0.07) | (0.06) | (1.79) | (0.08) |
| Regional CPI for apparel costs | 0.00 | 0.01 | 0.00 | 0.01 | 0.51 | -0.03 |
|  | (0.01) | (0.01) | (0.01) | (0.01) | (0.32) | (0.02) |
| Regional CPI for education costs | 0.01 | 0.01 | 0.06* | -0.02 | 0.01 | 0.03 |
|  | (0.01) | (0.02) | (0.03) | (0.03) | (1.04) | (0.07) |
| Regional CPI for recreation costs | -0.00 | 0.01 | 0.01 | -0.05† | -2.97† | -0.02 |
|  | (0.01) | (0.02) | (0.02) | (0.02) | (1.56) | (0.03) |
| Regional CPI for transportation costs | 0.00 | -0.00 | -0.01 | 0.01 | -0.44 | 0.03 |
|  | (0.01) | (0.01) | (0.01) | (0.01) | (0.28) | (0.02) |
| Fair market rent for 2-bedroom ($2021) | -0.00 | -0.00 | 0.00 | -0.00 | 0.01 | -0.00 |
|  | (0.00) | (0.00) | (0.00) | (0.00) | (0.02) | (0.00) |
| Child age at the interview | 0.01 | 0.03 | -0.00 | -0.05 | -3.45* | -0.13 |
|  | (0.02) | (0.03) | (0.04) | (0.09) | (1.34) | (0.08) |
| Squared child age at the interview | -0.00 | -0.00 | -0.00 | 0.00 | 0.03** | 0.00 |
|  | (0.00) | (0.00) | (0.00) | (0.00) | (0.01) | (0.00) |
| Mother's age | 0.09* | 0.07** | 0.04† | -0.03 | -2.43 | -0.06 |
|  | (0.03) | (0.03) | (0.02) | (0.05) | (2.01) | (0.06) |
| Squared mother's age | -0.00† | -0.00† | -0.00 | 0.00 | 0.02 | 0.00 |
|  | (0.00) | (0.00) | (0.00) | (0.00) | (0.02) | (0.00) |
| High school degree | -0.03 | -0.15* | 0.08 | -0.08 | -1.37 | 0.00 |
|  | (0.10) | (0.07) | (0.09) | (0.15) | (6.59) | (0.15) |
| Some College | -0.05 | -0.18† | 0.00 | 0.02 | -0.04 | -0.07 |
|  | (0.09) | (0.09) | (0.11) | (0.14) | (7.33) | (0.16) |
| BA or higher | -0.21 | -0.34* | -0.01 | 0.09 | -2.64 | -0.46 |
|  | (0.17) | (0.15) | (0.12) | (0.19) | (7.92) | (0.37) |
| Married | 0.03 | -0.15 | -0.01 | 0.23 | 3.03 | 0.02 |
|  | (0.12) | (0.16) | (0.10) | (0.15) | (4.41) | (0.30) |
| Urban | -0.01 | 0.08 | 0.04 | 0.10 | -3.56 | 0.16 |
|  | (0.07) | (0.14) | (0.12) | (0.17) | (5.93) | (0.45) |
| Urban-cluster | -0.11 | -0.04 | -0.26 | -0.10 | 3.32 | 0.19 |
|  | (0.11) | (0.11) | (0.20) | (0.19) | (6.28) | (0.56) |
| Family of three | -0.05 | -0.16 | -0.10 | -0.05 | 11.43 | 1.22*** |
|  | (0.06) | (0.13) | (0.19) | (0.15) | (8.10) | (0.30) |
| Family of four | 0.02 | -0.10 | 0.01 | -0.11 | 16.83* | 0.93** |
|  | (0.06) | (0.12) | (0.20) | (0.22) | (7.40) | (0.29) |
| Family of five | 0.07 | 0.01 | -0.17 | 0.06 | 11.78 | 0.79** |
|  | (0.07) | (0.13) | (0.15) | (0.16) | (7.54) | (0.26) |
| Family of six or more | 0.15 | 0.04 | -0.06 | 0.10 | 11.39 | 1.16*** |
|  | (0.11) | (0.15) | (0.18) | (0.17) | (7.99) | (0.23) |
| Received Medicaid | -0.10† | -0.08 | 0.12 | -0.09 | 1.59 | 0.31 |
|  | (0.05) | (0.08) | (0.10) | (0.09) | (4.15) | (0.31) |
| N | 1250 | 1250 | 1300 | 1300 | 1250 | 1300 |
| Notes: All estimates are weighted, except for sample sizes. † p<0.1, * p<0.05, ** p<0.01, *** p<0.001. Data come from wave 3 and wave k in the ECLS-B. | | | | | | |

**Figure S1.** Residuals from a Regression of the Thrifty Food Plan Price on Control Variables


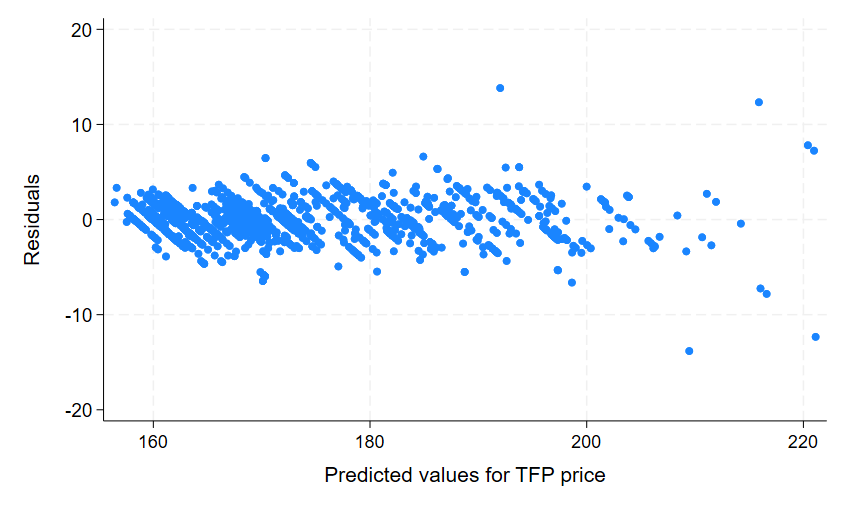


Notes: The figure shows residuals from a regression model of the TFP price on all control variables, including child and year fixed effects, state-level economic and policy characteristics, local prices of other goods, and child or family level characteristics. Data come from wave 3 and wave k in the ECLS-B (N=1300).

**Figure S2.** 35 market groups in Quarterly Food-at-Home Price Database (Reprinted from Todd et al., 2010).


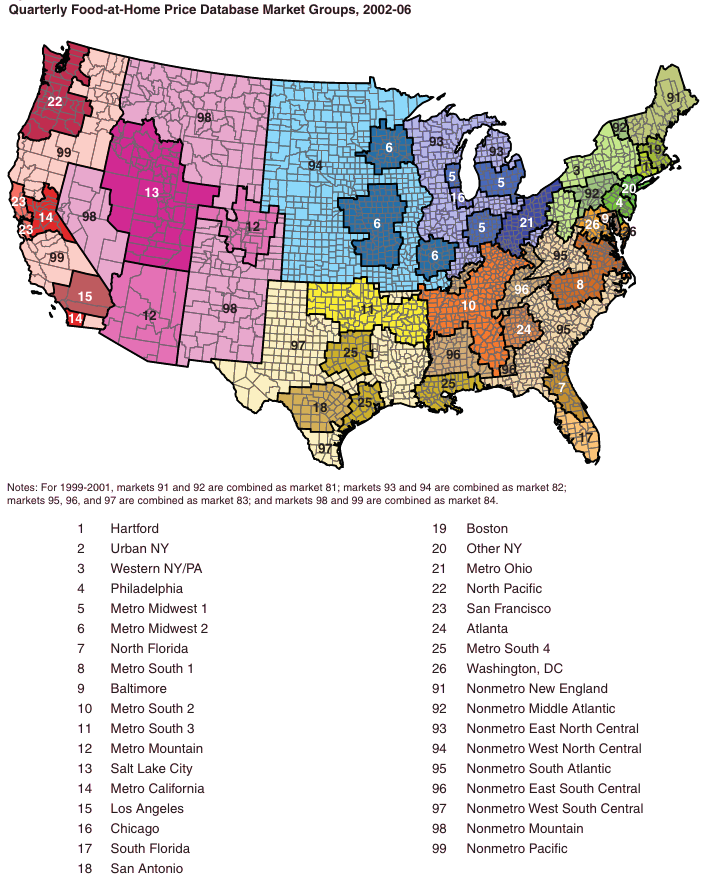


Notes: This figure is not subject to copyright.

Section A. Additional Details of the Methods

*Why the ECLS-B dataset is particularly suitable for this study:*

The Early Childhood Longitudinal Study-Kindergarten cannot be used, since it does not collect information on state of birth (or residence) in early childhood. The Future of Families and Child Wellbeing Study is another relevant data, but it represents children born in 1998 to 2000 only in U.S. 20 cities (15 states). Since my empirical strategy exploits the variation in SNAP purchasing power and maximum EITC across place (state or market group), having only 15 states will limit the amount of variations I can use. Panel Study of Income Dynamics-Child Development Supplement 2014 is also a nationally representative survey that collected reading and math scores. However, it collected development outcomes among a subsample (N=1498) and the sample size becomes even smaller when it is restricted to certain groups, limiting precision. Thus, these datasets are less preferable than the ECLS-B.

*Additional details on the construction of the SNAP purchasing power measure:*

Gregory and Coleman-Jensen (2013) developed a method to create a single price estimate of TFP for each market group and quarter. The first step is to map the individual QFAHPD food categories into a TFP food category (in most cases, a TFP food category consists of multiple QFAHPD food categories). The second step is to compute the price of each TFP food group. To do so, they use a weighted average of the quarterly prices for the QFAHPD foods within a TFP food category, where the weights are yearly national expenditure shares for the QFAHPD food in the TFP category. By averaging the quarterly price of TFP food categories across four quarters and then aggregating the TFP prices for all food groups, a single estimate of total TFP price can be calculated by market groups and years (see Gregory, & Coleman-Jensen (2013) and Bronchetti et al. (2019) for further details).

There are two versions of QFAHPD data. Version 1 of the QFAHPD (QFAHPD-1) contains prices for 52 food groups in 1999-2006 and version 2 of the QFAHPD (QFAHPD-2) contains prices for 54 food groups in 2004-2010. Since the SNAP purchasing power comes from 2003-2007 in my sample, I used both versions. To combine the two versions, I follow Bronchetti et al. (2019) and estimate the average ratio of the price in QFAHPD-1 to the price in QFAHPD-2 for 2004-2006 in each market group. Then, to put all price data into same units, I divide the price for 1999-2003 by this ratio. See this website for further details on QFAHPD-1 and QFAHPD-2: https://www.ers.usda.gov/data-products/quarterly-food-at-home-price-database/

*Additional explanations of using assessment timings as sources of variation in SNAP purchasing power and maximum EITC benefits:*

The variation in SNAP purchasing power and maximum EITC benefits may also come from different assessment timings. For instance, children who were assessed in 2005 and 2006 in wave 3 and wave k, respectively, are assigned SNAP purchasing power from 2004 and 2005 in each wave, while those assessed in 2005 and 2007 are assigned SNAP purchasing power from 2004 and 2006. Based on my consultation with the ECLS-B’s data team, the assessment timing was determined solely by a family’s availability and there is no record of field interviewers scheduling the assessments by nonrandom child or family characteristics. Moreover, descriptive analysis that compares demographic and socioeconomic characteristics by different assessment timing groups demonstrates that there are no statistically significant differences across those groups for most characteristics (see Table S10).
